# Screening of immunogenic proteins and evaluation of vaccine candidates against *Mycoplasma synoviae*

**DOI:** 10.1038/s41541-023-00721-y

**Published:** 2023-08-15

**Authors:** Guihua Zhang, Lejiabao Han, Zewei Li, Yifei Chen, Quan Li, Shifeng Wang, Huoying Shi

**Affiliations:** 1 College of Veterinary Medicine, Yangzhou University, Yangzhou, 225009 Jiangsu China; 2 Jiangsu Co‐Innovation Center for the Prevention and Control of Important Animal Infectious Diseases and Zoonoses, Yangzhou, China; 3 Department of Infectious Diseases and Immunology, College of Veterinary Medicine, University of Florida, Gainesville, FL 32611‐0880 USA; 4 Joint International Research Laboratory of Agriculture and Agri‐Product Safety, Yangzhou University (JIRLAAPS), Yangzhou, China

**Keywords:** Protein vaccines, Protein vaccines, Recombinant vaccine

## Abstract

*Mycoplasma synoviae* (*M. synoviae*) is a serious avian pathogen that causes significant economic losses to chicken and turkey producers worldwide. The currently available live attenuated and inactivated vaccines provide limited protection. The objective of this study was to identify potential subunit vaccine candidates using immunoproteomics and reverse vaccinology analyses and to evaluate their preliminary protection. Twenty-four candidate antigens were identified, and five of them, namely RS01790 (a putative sugar ABC transporter lipoprotein), BMP (a substrate-binding protein of the BMP family ABC transporter), GrpE (a nucleotide exchange factor), RS00900 (a putative nuclease), and RS00275 (an uncharacterized protein), were selected to evaluate their immunogenicity and preliminary protection. The results showed that all five antigens had good immunogenicity, and they were localized on the *M. synoviae* cell membrane. The antigens induced specific humoral and cellular immune responses, and the vaccinated chickens exhibited significantly greater body weight gain and lower air sac lesion scores and tracheal mucosal thicknesses. Additionally, the vaccinated chickens had lower *M. synoviae* loads in throat swabs than non-vaccinated chickens. The protective effect of the RS01790, BMP, GrpE, and RS00900 vaccines was better than that of the RS00275 vaccine. In conclusion, our study demonstrates the potential of subunit vaccines as a new approach to developing *M. synoviae* vaccines, providing new ideas for controlling the spread of *M. synoviae* worldwide.

## Introduction

*M. synoviae* is a significant avian pathogen that can cause acute or chronic diseases in poultry, including synovitis and airsacculitis. These conditions can lead to growth retardation and a reduction in egg production^1^. Previous studies have reported a higher global occurrence rate (38.4%) for *M. synoviae* compared to *Mycoplasma gallisepticum* (MG)^2^. Although *M. synoviae* may not directly cause poultry deaths, it can result in mixed infections with other avian pathogens such as Avian reovirus (ARV)^3^, Infectious laryngotracheitis virus (ILTV)^4^, Newcastle disease virus (NDV)^5^, *Escherichia coli* (*E. coli*), and Infectious bronchitis virus (IBV)^6^. This can lead to more severe diseases and result in significant economic losses during intensive poultry production worldwide.

Three strategies are commonly implemented on farms to control pathogenic avian *M. synoviae*, including maintaining flocks free of infection, antibiotic control, and immunization. However, maintaining flocks free of infection may be cost-prohibitive for many farms in various countries and regions^7^. Antibiotics can be very useful in preventing *M. synoviae* clinical signs and lesions, as well as economic losses, but their overuse can lead to antibiotic resistance^8^. Therefore, vaccination against *M. synoviae* could be a valuable long-term solution for commercial poultry production sites. The inactivated vaccine and live attenuated vaccine available in the market have made great contributions to the prevention and control of *M. synovia*. However, currently inactivated and available live attenuated vaccines have some drawbacks. The use of live vaccines requires chickens that test negative for antibodies due to their immunosuppressive nature^1^. Additionally, inactivated vaccines can be limited by the need for individual bird administration and local vaccine reactions^9^. Consequently, it is highly necessary to develop new-generation vaccines against *M. synoviae*.

The subunit vaccine strategy may provide a new option for the design of *M. synoviae* vaccines. In recent years, subunit vaccine development has been undertaken for other mycoplasma diseases^10,11^. However, developing subunit vaccines for *M. synoviae* has been extremely challenging due to a lack of understanding of its proteins. Only a few *M. synoviae* antigens, such as enolase^12^, pyruvate dehydrogenase complex E1 alpha and beta subunits (PDHA and PDHB), dihydrolipoamide dehydrogenase (PdhD)^13^, P35^14^, and NADH oxidase (NOX)^15^, have been reported. While these studies have contributed to understanding the biological function and pathogenesis of *M. synoviae* proteins, the immune response levels and protective immunity effects of these proteins have not been validated using animal models. Previous studies attempted to identify the immunogenic protein of *M. synoviae* using N-terminal sequencing. However, only a limited number of proteins were identified, and these proteins were not subsequently expressed or verified for protective efficacy^16^. Immunoproteomics and reverse vaccinology (RV) are two powerful methods used for identifying vaccine targets^17,18^. With the development of these techniques, large-scale screening of immunogenic proteins has become more convenient. These approaches have been widely used in the discovery and development of new vaccines for infectious diseases^19,20^.

To address these challenges, the present study aimed to identify potential immunogenic proteins of *M. synoviae* and to test their protective efficacy in a preliminary manner. Immunoproteomics and reverse vaccinology approaches were used to screen for immunogenic proteins, resulting in the identification of 24 potential vaccine candidates for *M. synoviae*. Among these candidates, RS01790 (a putative sugar ABC transporter lipoprotein), BMP (a substrate-binding protein of the BMP family ABC transporter), GrpE (a nucleotide exchange factor), RS00900 (a putative nuclease), and RS00275 (an uncharacterized protein) were selected for preliminary verification in a chicken model. The antigenic proteins identified in this study hold promise as vaccine candidates for *M. synoviae*, and may provide new insights for the development of *M. synoviae* vaccines.

## Results

### Identification of 24 potential vaccine candidates

**Fig. 1:**
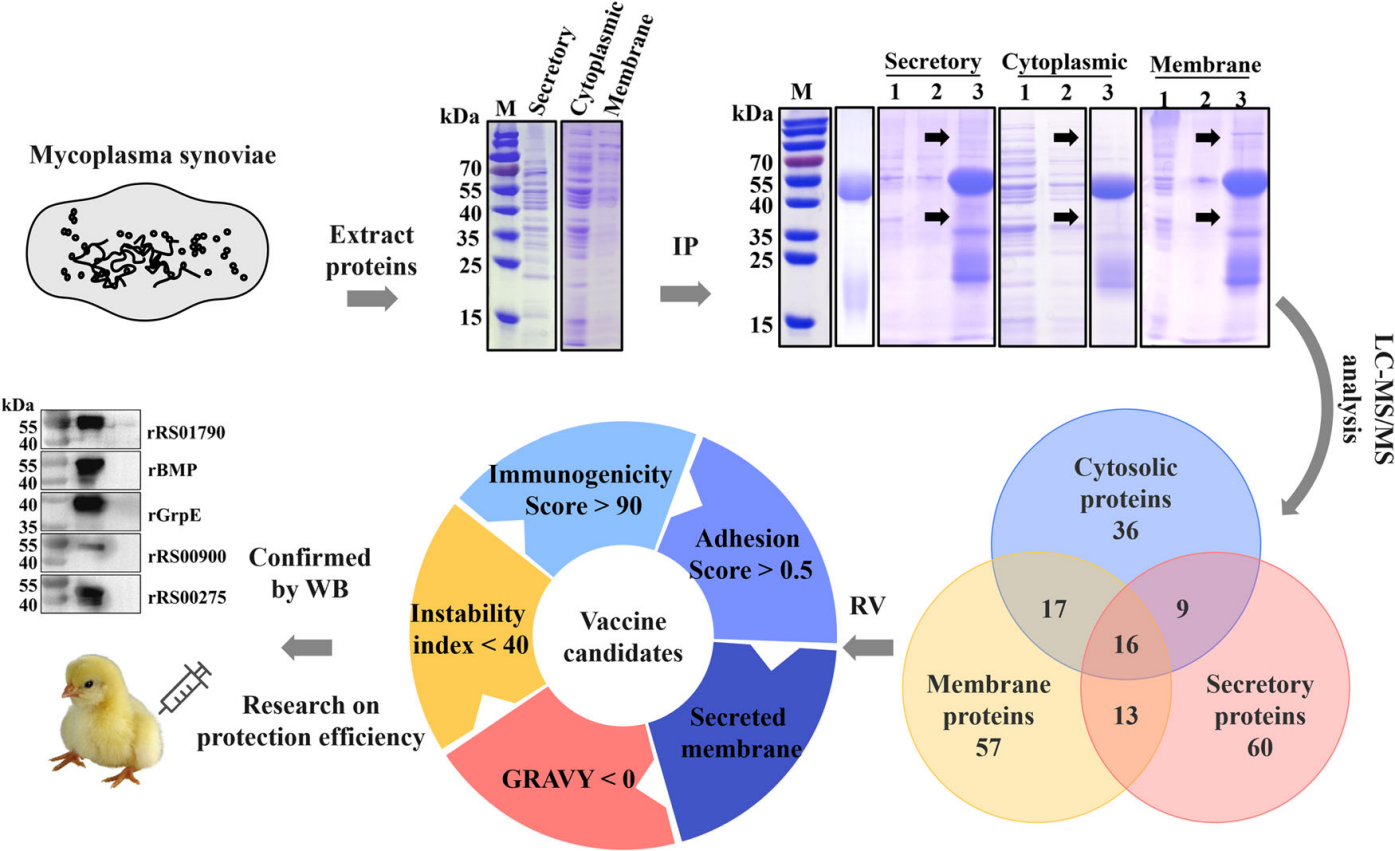
Designed workflow with the methodologies used for the prediction of vaccine. Extracted cytoplasmic, membrane, and secretory proteins of *M. synoviae* to complete immunoprecipitation with Purified hyperimmune sera from rabbits immunized with *M. synoviae*. Through mass spectrometry analysis, identified immunogenic proteins. Further screened these based on the antigenicity, adhesion capacity, instability index, grand average hydropathy (GRAVY), and number of transmembrane helices (TMHs). Finally, determined protein immunogenicity and immune protection efficiency through in vitro and in vivo experiments.

In this study, reverse vaccinology strategy was used to screen potential vaccine targets against *M. synoviae* following immunoproteomics analysis (Fig. 1). Purified hyperimmune sera from rabbits immunized with *M. synoviae* (Supplementary Fig. 1) and extracted cytoplasmic, membrane, and secretory proteins of *M. synoviae* were used for immunoprecipitation (Supplementary Fig. 2). Through immunoprecipitation and mass spectrometry analysis, a total of 208 immunogenic proteins were successfully identified (Supplementary Fig. 3). To estimate the antigenicity and adhesion capacity, all protein targets of *M. synoviae* were submitted to the Vaxign software. Among the 208 proteins, 90 had an antigenicity score greater than 90, 29 had an adhesion score greater than 0.5, and 28 satisfied both screening conditions. We gave priority to 26 secreted and membrane proteins, which are more likely to be immunogenic due to their exposure to the host’s immune cells. Subsequently, we used the same dataset of 26 secreted and membrane proteins to predict the instability index, grand average hydropathy (GRAVY), and number of transmembrane helices (TMHs).

The 24 potential vaccine candidates were selected, including two ABC transporters, two ABC transporter substrate‐binding lipoproteins, two Hemagglutinin, a putative endoglucanase, an DNA/RNA endonuclease, a nicotinate phosphoribosyl transferase, a single‐stranded DNA‐binding protein, an DegV‐like protein, an NADH oxidase (NOX), a 50 S ribosomal protein L10, a Translation elongation factor P, a YbhB/YbcL family Raf kinase inhibitor‐like protein, a purine‐nucleoside phosphorylase, a thiol peroxidase, a putative nuclease, a nucleotide exchange factor GrpE, and 5 hypothetical proteins (Table 1).

**Table 1:** Subcellular localization, adhesion and antigenicity score of *M. synoviae* proteins.

No.	Accession No.	Protein	Antigenicity score	Adhesion score	GRAVY	Instability index	TMHs	Location
1	WP154221545.1	Putative sugar ABC transporter	99	0.51	−0.225	27.14	0	M
2	WP011283299.1	BMP	98.9	0.516	−0.341	26.06	0	M, S
3	WP154221442.1	Putative nuclease	98.6	0.661	−0.462	28.38	1	M, S
4	WP154221360.1	Uncharacterized protein	98.2	0.58	−0.072	15.75	0	M, S
5	WP154221698.1	Uncharacterized protein	96.8	0.618	−0.387	36.73	2	M
6	WP154221661.1	Putative endoglucanase	94.2	0.556	−0.15	15.45	0	M
7	WP154221458.1	Hemagglutinin	92.8	0.707	−0.518	15.85	0	M
8	WP154221451.1	Uncharacterized protein	92.7	0.681	−0.34	31.26	0	S
9	WP154221765.1	Hemagglutinin	91.6	0.822	−0.336	18.83	0	M
10	WP154221558.1	P37-like ABC transporter substrate-Binding lipoprotein	91.5	0.538	−0.416	25.74	0	S
11	WP154221541.1	DNA/RNA endonuclease	91.4	0.657	−0.633	19.1	1	S
12	WP154221415.1	Nicotinate phosphoribosyltransferase	91.4	0.527	−0.418	26.59	1	M
13	WP154221707.1	Single-stranded DNA-binding protein	90.9	0.608	−0.574	36.4	0	M
14	WP154221436.1	DegV-like protein	90.9	0.583	−0.07	26.41	0	M
15	WP154221557.1	NADH oxidoreductase	90.9	0.577	−0.295	11.93	0	M
16	WP011283563.1	50 S ribosomal protein L10	90.9	0.573	−0.018	23.97	0	M
17	WP154221687.1	Uncharacterized protein	90.9	0.566	−0.275	21.1	0	M, S
18	WP154221338.1	Translation elongation factor P	90.9	0.55	−0.441	36.34	0	S
19	WP154221703.1	YbhB/YbcL family Raf kinase inhibitor-like protein	90.9	0.525	−0.458	23.9	0	M
20	WP011283588.1	Purine-nucleoside phosphorylase	90.9	0.519	−0.209	35.37	0	M
21	WP154221389.1	Thiol peroxidase	90.9	0.5	−0.271	27.89	0	M
22	WP154221527.1	Uncharacterized protein	90	0.625	−0.628	35.84	0	S
23	WP154221638.1	Putative ABC transporter ATP-binding protein	90	0.533	0.561	28.17	2	S
24	WP154221391.1	nucleotide exchange factor GrpE	90.9	0.452	−0.691	38.3	0	M, S, C

*C* Cytoplasmic, *M* Membrane, *S* Secretory

### Obtained highly pure recombinant antigen proteins

**Fig. 2:**
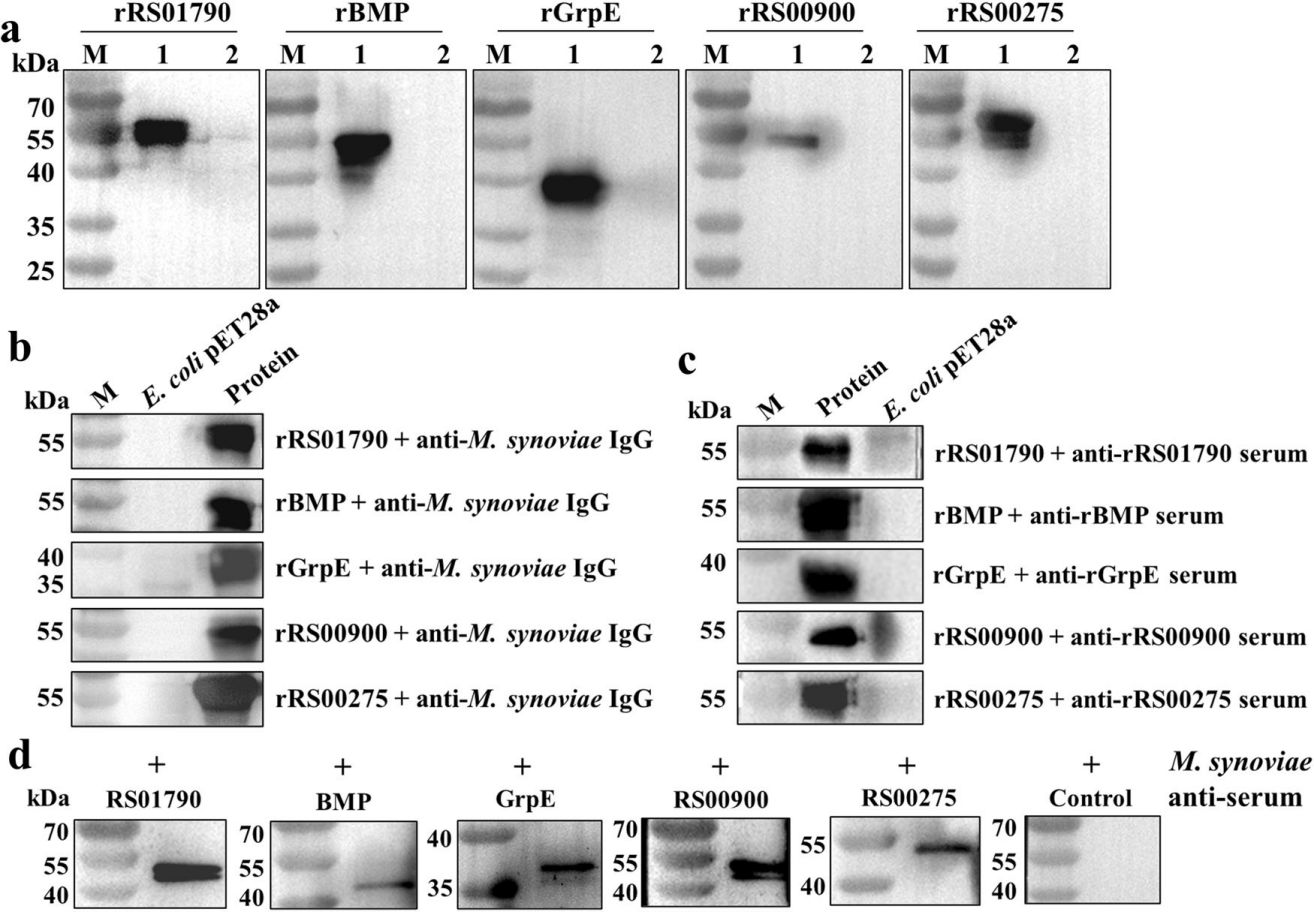
Expression and immunoreactivity of *M. synoviae* RS01790, BMP, GrpE, RS00900 and RS00275. **a** Western blot analysis of purified recombinant proteins using mouse anti‐His IgG. M: protein marker; Lane 1: recombinant proteins; Lane 2: total‐cell lysate of *E. coli* pET28a. **b** 10 μg of recombinant proteins were separated by SDS‐PAGE and then incubated with rabbit anti‐*M. synoviae* IgG. **c** 10 μg of recombinant proteins were separated by SDS‐PAGE and then incubated with the sera of mice immunized with rRS01790, rBMP, rGrpE, rRS00900, and rRS00275, respectively**. d** 10 μg *M. synoviae* cell lysates were separated by SDS‐PAGE and then incubated with sera from mice immunized with rRS01790, rBMP, rGrpE, rRS00900, and rRS00275, respectively.

Five antigen proteins involved in different families, including RS01790, BMP, GrpE, RS00900 and RS00275 were selected for preliminary verification randomly. They have more than 97% homology in the whole genome of 19 strains of *M. synoviae* (Supplementary Table 1). The results show that the artificial genes were successfully cloned into the pET28a (+) vector and the positive clones were designated as pET28a‐RS01790, pET28a‐BMP, pET28a‐GrpE, pET28a‐RS00900, pET28a‐RS00275 (Supplementary Fig. 4a). For the purification of recombinant proteins, the proteins were expressed with a histidine tag in *E. coli* BL21(Rosetta). The molecular weights were the correct size for RS01790 (~56 kDa), BMP (~49 kDa), GrpE (~34 kDa), RS00900 (~50 kDa) and RS00275 (~53 kDa), respectively, as evidenced in SDS‐PAGE (Supplementary Fig. 4b) and western blot (Fig. 2a) assays. The original blots are given in Supplementary Fig. 5.

### Five antigens are antigenic and immunogenic

As shown in Fig. 2b, rRS01790, rBMP, rGrpE, rRS00900, and rRS00275 reacted specifically with the anti‐*M. synoviae* IgG, but there was no obvious reaction with empty plasmid sample. The data suggested that all five proteins have good immunoreactivity with anti‐*M. synoviae* IgG. Additionally, the results indicated that five recombinant proteins can react specifically with antisera induced by themselves, respectively (Fig. 2c). Furthermore, according to the results, the five antisera against the rRS01790, rBMP, rGrpE, rRS00900, and rRS00275 reacted specifically with *M*. *synoviae* cells at the target band. However, there was no specific reaction between the sera of non‐immunized mice and *M*. *synoviae* cells (Fig. 2d). These results indicated that these five antigens were antigenic and immunogenic. They are potential *M. synoviae* antigen targets. The original blots are given in Supplementary Figs. 6–8.

### Five antigens could locate on *M. synoviae* membrane

**Fig. 3:**
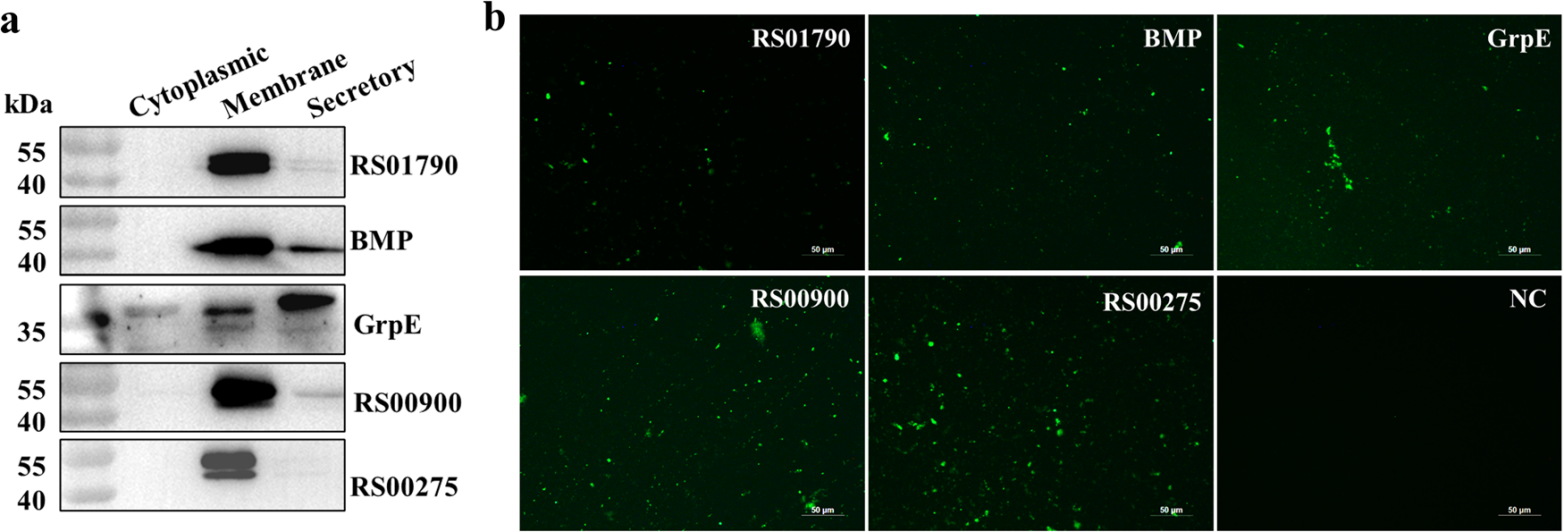
Surface distribution and localization of *M. synoviae* RS01790, BMP, GrpE, RS00900 and RS00275. **a** Western blot analysis of the distribution. **b** Suspension immunofluorescence assays. *M. synoviae* cells were incubated with mouse anti sera of five recombinant proteins, or non‐immunized mouse sera, followed by goat anti‐mouse IgG‐FITC antibody. NC Non‐immunized mouse sera control (Scale bars = 50 µm).

Western blot analysis was used to investigate the distribution of proteins in *M. synoviae*. The mouse antisera against rRS01790, rBMP, rRS00900, and rRS00275 reacted strongly with the membrane of *M. synoviae*, indicating their presence in the membrane. Additionally, the antisera against rBMP and rRS00900 also reacted with the secreted proteins of *M. synoviae*. GrpE was found in all the components of *M. synoviae*, with the highest proportion in secretory proteins and the lowest in cytoplasmic proteins (Fig. 3a). The original blots are given in Supplementary Fig. 9. The presence of GrpE in cytoplasmic, membrane, and secretory proteins of *Mycobacterium tuberculosis* validates the reliability of our experiment^21^. Additionally, specific bands corresponding to GroEL, a bacterial cytoplasmic reference protein, were detected in the whole bacterial protein fraction. However, GroEL bands were not detected in the extracted membrane and secreted protein fractions. This indicates that the extracted membrane proteins and secreted proteins do not contain cytoplasmic proteins such as GroEL (Supplementary Fig. 10).

To determine the surface localization of RS01790, BMP, GrpE, RS00900, and RS00275 in *M. synoviae*, suspension immunofluorescence assays were conducted using mouse anti-rRS01790, anti-rBMP, anti-rGrpE, anti-rRS00900, and anti-rRS00275 sera along with FITC-conjugated goat anti-mouse IgG. Positive staining of *M. synoviae* whole cells was observed when incubated with the five antisera against recombinant proteins, while no fluorescence staining was observed after incubation with non-immunized mouse sera (Fig. 3b). These results clearly demonstrate that RS01790, BMP, GrpE, RS00900, and RS00275 localize to the surface of *M. synoviae*. Subcellular localization results further confirmed the mass spectrometry sequencing results, where all five proteins were detected in the *M. synoviae* cell membrane fraction.

### Five antigens could adhere to DF-1 cells

**Fig. 4:**
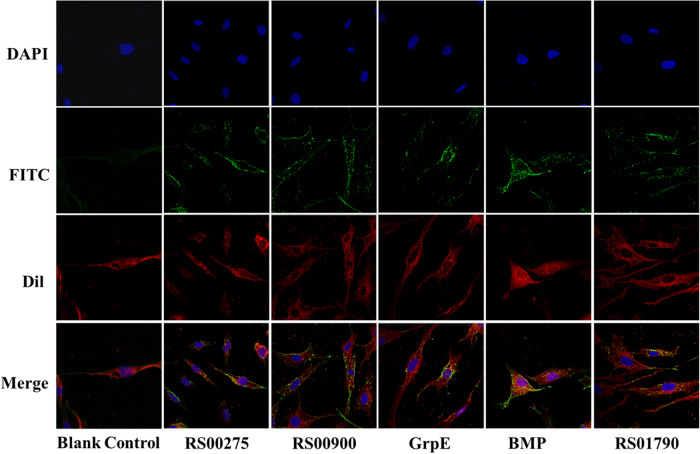
Adhesion of rRS01790, rBMP, rGrpE, rRS00900 and rRS00275 to DF‐1 cells. The protein was stained by mouse antisera and goat anti‐mouse IgG-FITC (green). Cellular nuclei and membrane were stained with DAPI (blue) and Dil (red), respectively.

Numerous adhesion proteins have demonstrated protective effects and are considered potential vaccine candidates^22^. The adherence of rRS01790, rBMP, rGrpE, rRS00900, and rRS00275 proteins to DF-1 cells was determined through indirect immunofluorescence. In comparison to cells without protein treatment, cells incubated with these five antigenic proteins exhibited green fluorescence on the surface (Fig. 4). These results indicated that the five antigenic proteins adhere to DF-1 cells.

### Immunization of chickens with antigens elicits a potent adaptive immune response

**Fig. 5:**
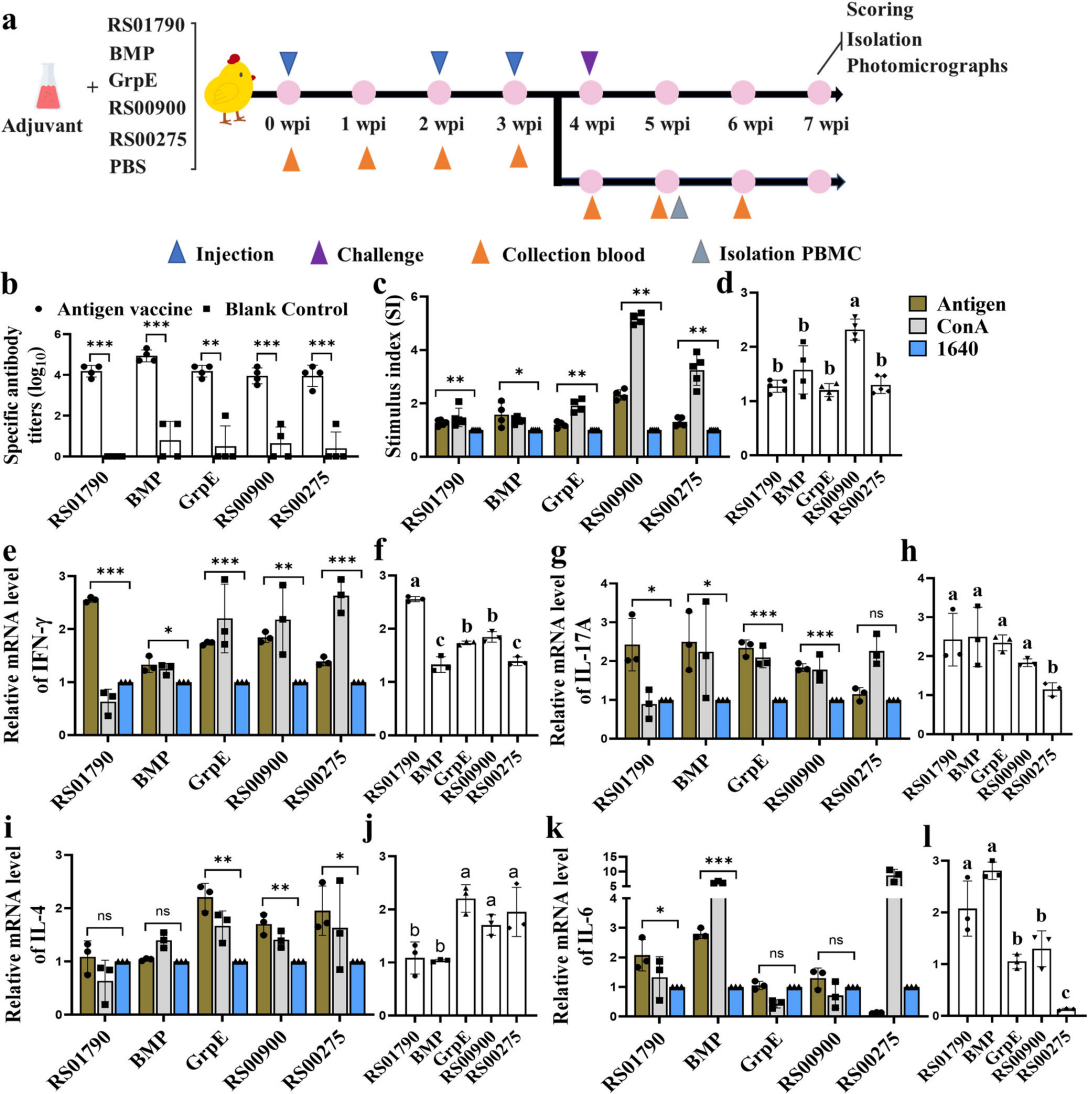
Scheme of immunization and challenge regimen and immune responses elicited by antigen proteins. **a** Schematic diagram of the immunization and challenge experiment in chicken. The chickens immunized intramuscularly three times with PBS or rRS01790, rBMP, rGrpE, rRS00900, and rRS00275 subunit vaccines. They were then challenged with *M. synoviae* rSC0200 strain (9 × 10^7^ CCU) one week after the final immunization. The first immunization was administered when the chickens were one day old (0 weeks post immunization (0 wpi)), followed by booster immunizations at 15 days and 22 days of age. At 29 days old, the chickens were challenged with *M. synoviae* and euthanasia was performed at 50 days old (7 wpi). The experiments were performed in triplicate. **b** IgG titers in sera were determined by ELISA and represented as dilution titers. **c**, **d** The proliferation response of chicken peripheral blood lymphocyte cells was detected by the CCK‐8 assay. The samples of the blood came from the randomly selected 3 chickens in each group of RS01790, BMP, GrpE, RS00900 and RS00275 and PBS. **e**–**l** mRNA expression levels of IFN‐γ (**e**, **f**), IL‐17 A (**g**, **h**), IL‐4 (**i**, **j**), IL‐6 (**k**, **l**) in lymphocyte cells were quantified. Values within a column with different lowercase superscripts (**a**, **b**, **c**) are significantly different (*p* < 0.05) in figures d, f, h, and l. Statistical analysis was performed using the Mann–Whitney U test (***p < 0.001, **p < 0.01, *p < 0.05, ns *p* > 0.05). Error bar, mean ± S.D.

The specific antibody levels in sera were determined using ELISA. As early as two weeks after primary vaccination, specific antibodies against the antigen proteins could be detected, and their levels increased rapidly upon boost vaccination (Supplementary Fig. 6). RS01790, BMP, GrpE, RS00900 and RS00275 were found to induce total IgG antibody titers of 1 × 10^4.2^, 1 × 10^4.9^, 1 × 10^4.1^, 1 × 10^4^, 1 × 10^4^, respectively, three weeks after the final immunization (Fig. 5b).

Cell-mediated immune response is crucial for clearing mycoplasma infections and may have a significant role in vaccine-mediated protective immune response^20^. To assess the cellular immune response triggered by subunit vaccines immunization, the lymphocyte proliferation and cytokine production of immunized chicken were analyzed using peripheral blood mononuclear cells (PBMCs). The results demonstrated that PBMCs stimulation with antigens resulted in significantly higher proliferation levels compared to the control group (cells without protein stimulation). Moreover, the lymphocyte proliferation induced by RS00900 was significantly higher than that observed in other immune groups (Fig. 5c, d).

Stimulation of cells with the antigens RS01790 and BMP in vitro increased the levels of IFN‐γ, IL‐17 A, and IL‐6. Stimulation with GrpE and RS00900 induced significant increases in the levels of IFN‐γ, IL‐17 A and, IL‐4. However, stimulation with RS00275 only increased the mRNA level of IFN‐γ and IL‐4. The IFN‐γ upregulation induced by RS01790 protein stimulation was the most significant compared with other immune groups (Fig. 5e, f). Only RS00275 stimulation did not cause an increase in the of level IL‐17 A, and other immune groups had no significant difference (Fig. 5g, h). RS01790 and BMP stimulation did not cause significantly upregulate IL‐4 (Fig. 5i, j). After stimulation with GrpE, RS00900 and RS00275, the levels of IL‐4 increased, but the levels of IL‐6 remained unchanged (Fig. 5k, l). Overall, RS01790, BMP, GrpE, and RS00900 induced Th1, Th2 and Th17 cellular immune responses, while RS00275 induced Th1 and Th2 cellular immune responses but did not induce Th17. These data indicated that immunization with the five subunit vaccines can activate cellular immunity in chickens, respectively. Furthermore, based on the results of average daily weight gain (ADWG) from day 0 (one day old) to days 28 (29 days old), the immunization with the five subunit vaccines did not affect the growth of chickens (Supplementary Fig. 12).

### Vaccine candidates protect against *M. synoviae* infection

**Fig. 6:**
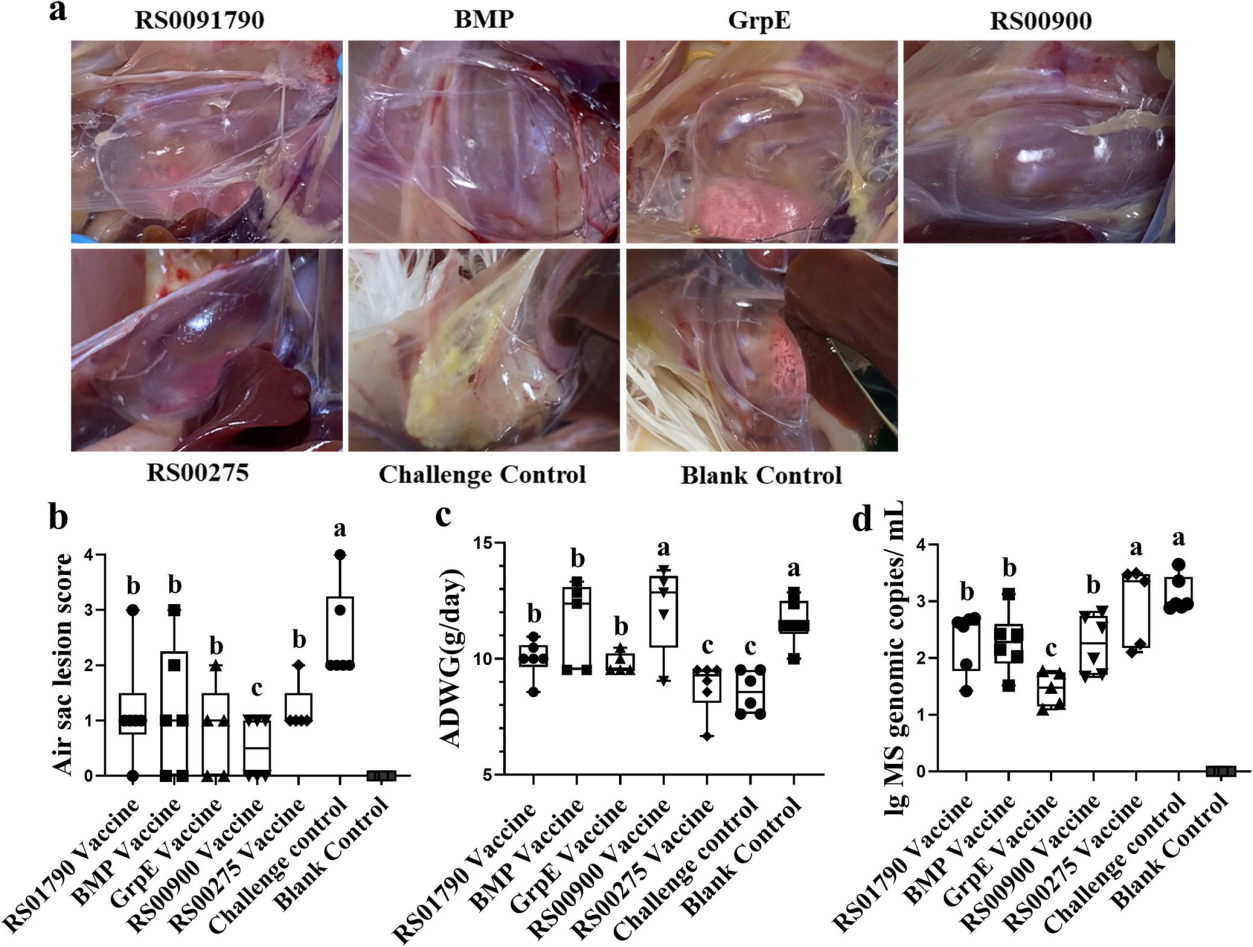
Gross air sac lesions, *M. synoviae* loads and ADWG in SPF chicken at 21 days post infection. **a** Appearance of the air sac in chicken after 3 weeks of infection. **b** Air sac lesion scores in chicken at 3 weeks after challenge among groups on a 5-point scale from 0 to 4 (0, No airsac lesion was observed; 1, Slight cloudiness of the air sac membrane were found; 2, Air sac membrane was slightly thick and usually presented small accumulations of cheesy exudates; 3, Air sac membrane was obviously thick and meaty in consistency, with large accumulations of cheesy exudates in one air sac; 4, Lesions were observed the same as 3, but 2 or more air sacs were found.). **c** Results of average daily weight gain (ADWG) in chickens after challenge. **d** Microbial loads of *M. synoviae* in chicken throat swab measured by qPCR method after being challenged with rSC0200 strain. Statistical analysis was performed using the Mann–Whitney U test. Error bar, mean ± S.D. Values within a column with different lowercase superscripts (a, b, c) are significantly different (*p* < 0.05) in (**b**–**d**).

There were only mild air sac lesions in the groups inoculated with the five subunit vaccines. However, chickens from the challenge control group showed significant higher lesion scores than subunit vaccine groups (Fig. 6a, b). The lesion score of the RS00900 immunization group was significantly lower than that of the other vaccinated groups. After challenge infection (29–50 days old), ADGW of the RS01790, BMP, GrpE, and RS00900 immunization groups was significantly higher compared to the challenge group. There was no significant difference between BMP and RS00900 immunization groups and the blank control group. However, there was no significant difference between the RS00275 immunization group and the challenge control group (Fig. 6c). The mean DNA copy number of positive samples in the subunit vaccine groups was significantly lower than in the challenge group, expect for the RS00275 immunization group. The *M. synoviae* load of the GrpE immunization group was significantly lower than that of the other vaccinated groups. No *M. synoviae* was detected in the blank control group (Fig. 6d).

**Fig. 7:**
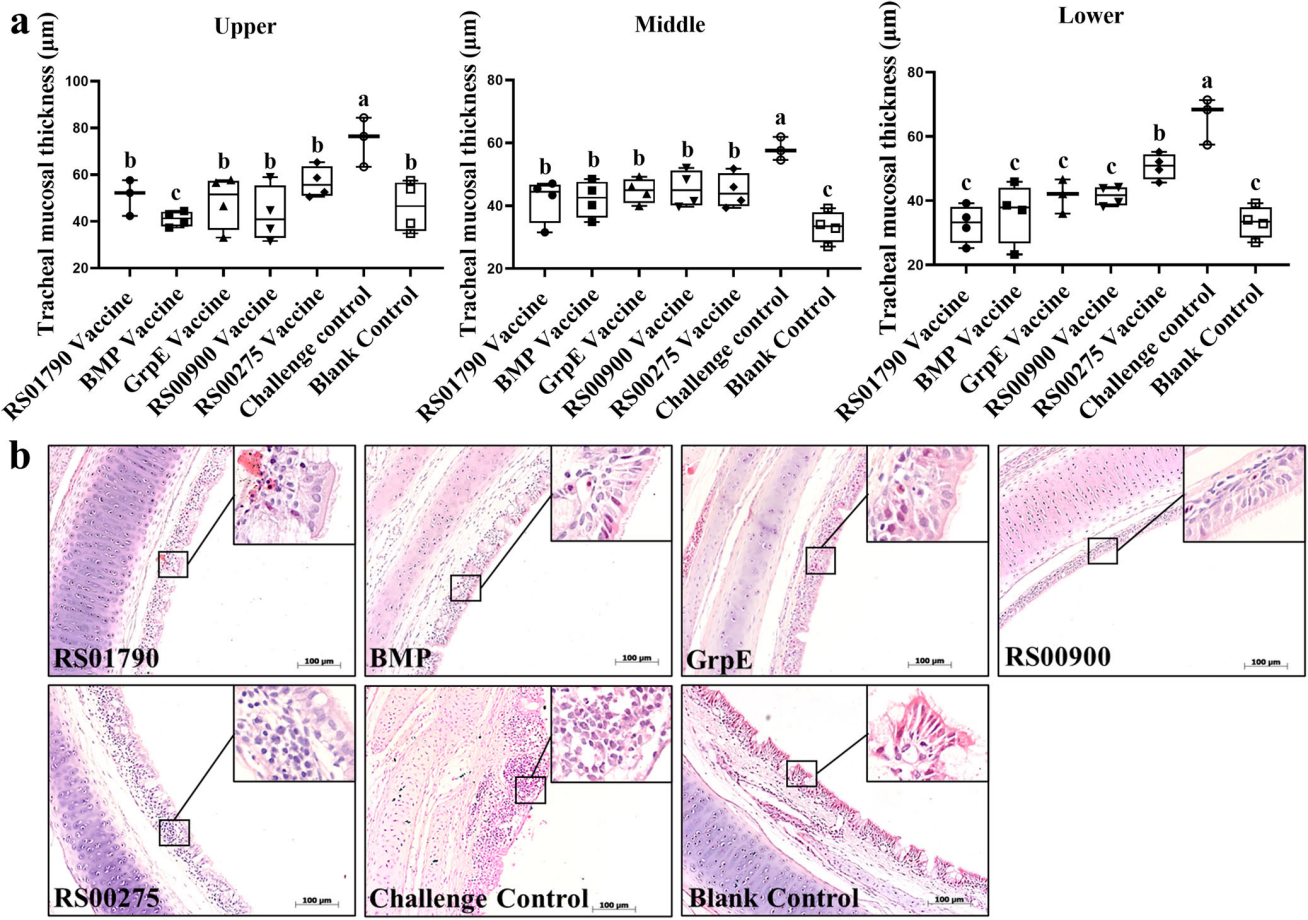
Pathological lesions of chicken tracheal mucosae tissue and mean tracheal mucosal thickness after *M. synoviae* challenge. **a** The mean tracheal mucosal thickness of the upper, middle and lower tracheas of the chicken on day 21 after challenge with *M. synoviae*. **b** Tracheal mucosae tissue of the experimental chickens was sectioned and stained with H&E on day 21 after challenge with *M. synoviae*. Scale bar = 100 µm. Statistical analysis was performed using the Mann–Whitney U test. Error bar, mean ± S.D. Values within a column with different lowercase superscripts (a, b, c) are significantly different (*p* < 0.05) in (**a**).

The mean tracheal mucosal thickness (micrometer, μm) of the upper, middle and lower tracheas of the chickens in the five subunit vaccine groups was significantly thinner than that of the challenge control group (Fig. 7a). The mean thickness of the upper tracheal mucosa in the BMP immunization group was significantly lower than that in the other groups. The mean thickness of the middle tracheal mucosa in the five vaccinated groups was significantly higher than that in the blank control group, with no significant difference observed among the vaccinated groups. In the lower trachea, the tracheal mucosa of the RS00275 group was significantly thicker than that of the blank control group, while there was no significant difference observed between the other vaccinated groups and the blank control group.

The pseudo‐stratified ciliated columnar epithelium of the tracheal mucosa, along with abundant simple intraepithelial mucous glands containing several goblet cells, was observed in the RS01790, BMP, GrpE, RS00900 vaccinated groups, as well as in the blank control group. Mild mucosal thickening resulting from several lymph follicular infiltrates was observed in tracheal mucosae of the RS00275 immunization group. In the tracheal mucosa of the chickens from the challenge group, neither ciliated columnar epithelium nor intraepithelial mucous glands were present, and there was lymphocytic infiltration. The columnar epithelium was replaced by simple squamous epithelium, and there was both diffuse infiltration and focal aggregation of lymphocytes (Fig. 7b). The results showed that RS01790, BMP, GrpE and RS00900 exhibited a better protective effect compared to RS00275 immunization group.

## Discussion

*M*. *synoviae* is one of the important *Mycoplasma* species infecting avians, and has been listed as the legally reported mycoplasma by the world organization for animal health (OIE)^23^. The difficulty in developing subunit vaccines for *M. synoviae* is attributed to insufficient knowledge about its proteins. Identification of antigens is very important and urgent for the development of effective vaccine candidates against *M*. *synoviae*. Therefore, the purpose of this study was to identify new *M*. *synoviae* antigens as potential vaccine candidates.

Immunoproteomics and RV have emerged as high‐throughput methods for screening vaccine candidates^17,18^. Currently, there is no systematic study on screening immunogenic proteins of *M*. *synoviae*. In this study, a combined approach of immunoproteomics and bioinformatics analysis was employed to identify new vaccine candidates in *M. synoviae*. Through prediction, 24 potential vaccine candidates, including membrane and secretory proteins, were identified. Among these proteins, ATP‐binding cassette (ABC) transporters are essential proteins found in nearly all living organisms^24^ and have been identified as potential protective antigens^25^. ABC transporters substrate-binding proteins are important targets for the development of antibacterial vaccines and therapies^26^. Immunization with BMP has been shown to provide protection against *Enterococcus faecium* in a murine infection model^27^. Nucleases in mycoplasmas are considered to play a crucial role in growth and pathogenesis^28^. In *Mycoplasma hyopneumoniae*, a nuclease (Mhp597) was identified as an immunodominant antigen, using reverse vaccinology^29^. GrpE plays a significant role in the survival of *Mycoplasma* during cellular stresses such as heat shock and hypoxia. It is a well‐known HSP70 cofactor^30,31^. GrpE has demonstrated significant protection against *Ureaplasma urealyticum* in a mouse model^10^. The hypothetical protein RS00275, verified in this study, has not been reported previously. RS00275 may be one of the mucin-associated surface proteins (MASPs). The MASPs are glycosylphosphatidylinositol (GPI)-anchored glycoproteins that are encoded by a multigene family consisting of hundreds of members. Many MASP family members have multiple predicted MHC-I and MHC-II epitopes, making them valuable targets for vaccine development^32^. These five proteins have not yet been researched in *M*. *synoviae*. They were selected for preliminary immunogenicity and protective verification in this study.

Being highly conserved among prevalent *M*. *synoviae* strains worldwide, being expressed and antigenic during infection, and eliciting long‐term protective immunity in vivo were requirements for an effective vaccine immunogen^33^. The five antigens have more than 97% homology in the whole genome of 19 strains of *M. synoviae*. The multiple antibody sera of *M. synoviae* recognized five proteins, and the sera immunized with these five proteins recognized the whole cell of *M. synoviae*. This indicates that they are antigenic and immunogenic, making them potential antigen targets. Membrane and secreted proteins are likely to be more antigenic than cytoplasmic proteins, making them easier targets for researchers when selecting candidates for vaccine development^34,35^. These five antigens are localized on the cell membrane of *M. synoviae* and have shown the ability to adhere to host cells. Based on the above results, five antigens were used in subsequent experiments as subunit vaccines.

A high antibody titer represents that the host has been experienced a strong humoral immune response. In comparison to the blank group, the five subunit vaccines induced a higher level of antibodies in chickens. These results suggested that the antigens enhanced the production of antigen‐specific antibodies. IL‐4 and IL‐6 are kwon to play important role in humoral immunity^36^. In this study, the expression of Th2‐type cytokines (IL‐4 and IL‐6) increased following stimulation with the five candidate vaccines. The consistent trend observed between Th2‐type cytokines and the specific antibody indicated that the five vaccines effectively activated robust humoral immune response.

It is widely known that cell‐mediated immunity, such as antigen‐specific Th1 and Th17 cells, plays a crucial role in preventing mycoplasma infections. IFN‐γ, primarily produced by Th1 cells, is an important mediator in this process^37^. Upon re-exposure to the immunizing antigens, lymphocytes from chickens immunized with RS01790, BMP, GrpE, RS00900, and RS00275 demonstrated high levels of IFN-γ production. IL-17, predominantly produced by Th17-type cells, is also critical in protection against mycoplasma infections^38^. The other four vaccinated groups, all showed an up‐regulation of IL‐17, except for RS00275 group, which showed no significant difference. The immune protection results indicated that the five vaccines were effective in reducing the damage to the air sac and tracheal mucosa caused by *M. synoviae*. However, the tracheal mucosa of the RS00275 immunization group still exhibited slight thickening due to lymphocyte infiltration. There was no significant difference in average daily weight gain (ADWG) from 29 to 50 days old and the bacterial load of *M. synoviae* in the throat between the RS00275 immunization group and the challenge control group. In general, the protective effect of RS01790, BMP, GrpE, and RS00900 vaccines was superior to that of RS00275.

In summary, our study aimed to identify protein candidates of *M. synoviae* for vaccine development using immunoproteomics and reverse vaccinology methodologies. Protein candidates were screened based on their antigenicity, adhesion probability, localization, instability index, and Grand average of hydropathicity (GRAVY). We predicted 24 antigens that have not been previously described as vaccine candidates in *M. synoviae*. The immunogenicity of five representative proteins, namely RS01790 (lipoprotein, putative sugar ABC transporter), BMP (BMP family ABC transporter substrate‐binding protein), GrpE (nucleotide exchange factor), RS00900 (putative nuclease) and RS00275 (Uncharacterized protein), was evaluated in vitro and in vivo. In a chicken model, RS01790, BMP, GrpE and RS00900, when administered with Montanide TM ISA 206 VG adjuvant, significantly reduced weight loss, air sac lesion scores, tracheal mucosal thicknesses, and the *M. synoviae* colonization in the larynx. The results indicated that RS01790, BMP, GrpE, RS00900 and RS00275 provided varying degrees of protection against *M. synoviae* challenge. This suggests that a vaccine based on a recombinant protein can effectively protect chickens from *M. synoviae* infections. Our study demonstrated the potential of using subunit vaccines and provides new insights for the development of *M. synoviae* vaccines. These vaccine candidates may be further explored in vaccine strategies against the *M. synovia*.

## Methods

### Animals and ethics statement

Female six-week-old BALB/c mice were purchased from the Comparative Medicine Center, Yangzhou University (Jiangsu, China). Female two-month-old New Zealand white rabbits (Shanghai Songlian Experimental Animal Farm, Shanghai, China). The specific pathogen-free (SPF) chicken embryos were purchased from Nanjing Biology Medical Factory, Qian Yuan-hao Biological Co., Ltd. The chickens used in this study were obtained by SPF chicken embryos in our laboratory. All animals were housed in conventional animal facilities with water and food ad libitum and were monitored at least twice daily. All animal experiments were performed at Yangzhou University with the approval of the Jiangsu Administrative Committee for Laboratory Animals (permission number SYXK (SU) 2021‐0026, SYXK (SU) 2017‐0044, SYXK (SU) 2021‐0027). The procedures conformed to the guidelines of Jiangsu Laboratory Animal Welfare and Ethics, following international law.

### Bacterial and cells

*M. synoviae* strain rSC0200 was isolated from a chicken and stored in our laboratory. rSC0200 strain was grown in the Frey Medium Modified Base (Solarbio, Beijing, China) supplemented with 10% horse sera, 0.1 g/L nicotinamide adenine dinucleotide (NAD), 0.1 g/L cysteine hydrochloride hydrate, 1,000,000 IU penicillin G (Shenggong, Shanghai, China)^13^. *Escherichia coli* (*E. coli*) strains DH5α and BL21(Rosetta) (Tiangen, Beijing, China) were used to clone or express the genes with the His-tag pET-28a (+) vector, which were cultured in Luria-Bertani (LB) broth or on solid media containing 1.5% agar with 50 μg/mL of kanamycin after being transformed with the recombinant expression vectors. The DF-1 chicken embryo fibroblast cells were stored in our laboratory. DF-1 cells were cultured at 37 °C in an atmosphere of 5% CO_2_. And Dulbecco’s Modified Eagle Medium (DMEM; Hyclone, Logan, UT, USA) with 10% fetal bovine sera (FBS; Gibco), 100 IU/mL of penicillin, and 100 μg/mL of streptomycin (Shenggong, Shanghai, China) were used.

### Indirect ELISA assays

An Indirect Enzyme-linked immunosorbent assay (ELISA) was performed to determine the antibody titers against *M. synoviae* whole cells and recombinant protein^39^. Microtiter plates were coated with sonicated protein antigens of *M. synoviae* (0.1 μg/mL) or recombinant protein (0.5 μg/mL) in 0.1 M sodium carbonate buffer (pH 9.6) and incubated overnight at 4 °C. The plates were then washed three times with phosphate buffer containing 0.05% Tween 20 (PBST). After incubating with blocking buffer (5% skim milk in PBST), the wells were incubated with serum diluted in PBST (ranging from 1∶1000 to 1∶128,000) at 37 °C for 2 h. Subsequently, 100 μL of goat anti-rabbit or goat anti-mouse IgG-HRP (H + L) antibody (diluted at 1∶5000) was added and incubated at 37 °C for 90 min (min). The color reaction was developed by the adding 100 μL of TMB (Solarbio, Beijing, China) and allowed to proceed for 15 min, followed by the addition of 50 μL of 2 M H_2_SO_4_ to stop the reaction. Finally, the optical density (OD) was measured at 450 nm in an automatic microplate reader. A sample was considered positive when the ratio of the positive value (P) to the negative value (N) was greater than 2.1 (P/N > 2.1).

### Western blot assays

Purified recombinant proteins, *M. synoviae* whole cells, or *M. synoviae* fractions were solubilized in sample buffer (50 mM Tris-HCl pH 6.8, 10% glycerol, 0.1%BPB, 1% 2-β-mercaptoethanol, and 2% SDS) and subjected to 12% SDS-PAGE. For western blot analysis, the protein was electrotransferred onto a PVDF membrane (Immun-Blot® PVDF, Bio-Rad) at 70 V for 90 min. The membranes were incubated in blocking buffer (5% skim milk in PBST) overnight at 4 °C. After washing with PBST, the membranes were primary incubated with the primary antibody for 2 h at 37 °C. Then, they were incubated with goat anti-rabbit or goat anti-mouse IgG-HRP (H + L) antibody (1∶5000 dilution) for 2 h at 37°C. The specific bands were detected using an enhanced chemiluminescence (ECL) substrate (Thermo Fisher). Equal volumes of Reagent A (luminol) and Reagent B (an enhancer) were gently added to the membrane surface after liquid mixing, and images were captured using a chemiluminescence detection system (Tianneng, Shanghai, China).

### Production of polyclonal antibodies against recombinant proteins and *M. synoviae* whole cells

The expressed proteins were appropriately diluted and mixed with an equal volume of QuickAntibody‐Mouse3W adjuvant (Biodragon, Suzhou, China). Female six‐week‐old BALB/c mice were intramuscularly immunized in the leg two times at a 2‐week interval, with each mouse receiving 50 μg of the immunogen. To generate rabbit sera against *M. synoviae*, three female New Zealand white rabbits were immunized subcutaneously. To generate rabbit sera against *M. synoviae*, three female New Zealand white rabbits were immunized subcutaneously. They received a prime immunization followed by three boosts on days 0, 14, 21, and 28. The vaccine formulation contained 800 μg of whole-cell *M. synoviae* combined with complete Freund’s adjuvant (Sigma, USA) on day 0, and incomplete Freund’s adjuvant on days 14, 21, and 28. One week after the final immunization, blood samples were collected from both immunized and non-immunized animals. The collected sera were isolated after centrifugation (3000 × *g*, 15 min), and their antibody titers were determined using an indirect ELISA assay as described above. The rabbit sera immunized with *M. synoviae* were dialyzed using Binding Buffer (0.15 M NaCl, 20 mM Na_2_HPO_4_, pH 7.0), and then purified by Protein A/G affinity chromatography according to the manufacturer’s instructions (Solarbio, Beijing, China)^40^. The IgGs were salted out with ammonium sulfate using a protocol previously described in ref. ^41^.

### Extraction protein of *M. synoviae*

Membrane and cytosolic protein fractions of *M*. *synoviae* were extracted following a previously described method with slight modifications^42^. The *M*. *synoviae* culture was collected by centrifugation and then resuspended in Tris-EDTA-buffered solution (50 mM Tris, 0.15 M NaCl, 1 mM EDTA, pH 8.0) containing 1 mM PMSF (protease inhibitor) and 2% (v/v) TX-114. The mixture was incubated at 4 °C for 60 min, followed by a 10 min incubation at 37 °C to induce phase separation. Subsequently, the mixture was centrifuged at 16,000 × *g* for 20 min. The upper aqueous phase was transferred to a second tube, and an equal volume of Tris-EDTA-buffered solution was added to replace it. Two and a half volumes of ethanol were then added to precipitate membrane lipoproteins, and the mixture was incubated overnight at −20 °C. Similarly, proteins from the aqueous phase were ethanol-precipitated overnight at −20 °C. The following day, the precipitated materials (lipoproteins from the TX-114 fraction and proteins from the aqueous phase) were recovered by centrifugation and resuspended in phosphate-buffered saline (PBS, pH 7.4) through brief sonication. Secretory proteins of *M*. *synoviae* were extracted following a previously described method with slightly modifications^43^. *M*. *synoviae* was cultured until it reached the late log phase (36 h), and then harvested by centrifugation at 16,000 × *g* for 40 min at 4 °C. The cells were washed twice and resuspended in PBS, followed by incubation at 37 °C for 2 h. After centrifugation under the same conditions, the supernatant was collected. The supernatants were filtered (0.45 μM) to remove potential impurities and subsequently concentrated using a molecular weight cut-off of 3.5 kDa. All proteins were analyzed by SDS-PAGE and western blot as described above. Western blot analysis was performed using a rabbit anti-*M. synoviae* antiserum (prepared in this study) and rabbit anti-groEL antibody (Abcom, ab90522; 1∶3000), followed by goat anti-rabbit IgG-HRP (H + L) antibody (Boster Biological Technology co.Itd, BA1054; 1∶5000).

### Immunoprecipitation

The immunoprecipitation (IP) assay using Protein A/G plus Agarose beads (Solarbio, Beijing, China) was conducted following the manufacturer’s instruction. Briefly, 50 μL aliquots of the beads were washed twice with 0.5 mL Binding Buffer (0.15 M NaCl, 20 mM Na_2_HPO_4_, pH 7.0). The beads were then mixed with the purified sera IgG and incubated at room temperature. After 30 min incubation, the beads were washed two times with Wash Buffer (0.15 M NaCl, 20 mM Na_2_HPO_4_, pH 7.0). The *M*. *synoviae* protein fractions were mixed with the anti-*M*. *synoviae* antibody-coated beads and incubated overnight at 4 °C. The antigen-antibody-beads complex was washed with the Wash Buffer and eluted with sample buffer (as described above) at 100 °C for 10 min. It was then centrifuged at 12,000 × *g* for 10 min, and the supernatant was collected for SDS-PAGE.

### LC‐MS/MS analysis

Protein digestion and Label‐free liquid chromatography‐tandem mass spectrometry (LC‐MS/MS) analysis were conducted by the Proteomics Platform of the GeneCreate (Wuhan, China). A clean blade was used to extract the specific strip of interest, which was then subjected to destaining and tryptic digestion following previously described protocols^44^. Briefly, the gel pieces were initially treated with 10 mM DTT at 56 °C for 30 min, followed by 55 mM IAM at room temperature for 30 min in a dark room. Next, 0.01 μg/μL trypsin was added based on the gel volume, and the mixture was incubated in an ice bath for 30 min. Subsequently, an appropriate volume of 25 mM NH_4_HCO_3_ PH 8.0 enzymatic hydrolysis buffer was added, and enzymatic hydrolyze was carried out overnight at 37 °C. The sample was dissolved using 10–20 μL of 0.2% TFA for desalting. Peptide samples were dissolved in 2% acetonitrile/0.1% formic acid and loaded onto both the C18 capture column and the C18 analytical column for gradient elution.

For Information Dependent Acquisition (IDA), the MS spectrum was scanned with an ion accumulation time of 250 millisecond, and the MS spectrum of 30 precursor ions was acquired with an ion accumulation time of 50 millisecond. MS1 spectrum was collected in the range of 350–1200 m/z, while MS2 spectrum was collected in the range of 100–1500 m/z. The precursor ion dynamic exclusion time was set to 15 s. The raw MS/MS files obtained from the mass spectrometer were submitted to ProteinPilot software for data analysis. obtained from the mass spectrometer were submitted to ProteinPilot software for data analysis.

### Bioinformatics analysis

The selected proteins from the previous LC‐MS/MS results were tested for immunogenicity and adhesion probability using the Vaxign 2.0 server (http://www.violinet.org/vaxign2.)^45^. Vaxign 2.0 employs a genomic feature-based method for predicting vaccine targets in the reverse vaccinology platform. The adhesion probability threshold was set at 0.5, and the immunogenicity threshold was set at 90 to predict proteins with probable antigenic and non‐antigenic properties^46^. The Protparam server (https://web.expasy.org/protparam/.) was utilized to calculate the physicochemical parameters of the proteins construct, including the instability index and grand average hydropathy (GRAVY)^47^. The number of transmembrane helixes in a protein is significant criterion for vaccine development, as they are relevant in the process to clone, express, and purify proteins. TMHMM 2.0 server (https://services.healthtech.dtu.dk/services/TMHMM-2.0/) was used to search for transmembrane helices in the proteins^48^. Results indicating exp number of AAs in TMHs (ExpAAs) < 18 and a maximum of two transmembrane helices (TMHs) were selected for further analysis.

### Expression of recombinant antigenic proteins

The sequence of *RS01790* (WP_154221545.1), *bmP* (WP_011283299.1), *grpE* (WP_154221391), *RS00900* (WP_154221442.1) and *RS00275* (WP_154221360.1) genes were optimized to make them suitable for the *E. coli* expression system, and TGA was changed to TGG (Wuhan GeneCreate Biological Engineering Co., Ltd)^49^. The optimized gene was synthesized and constructed into the expression vector pET28a. The vectors pET28a-RS01790, pET28a-BMP, pET28a-GrpE, pET28a-RS00900, and pET28a-RS00275 were introduced into *E. coli* strain BL21 (Rosetta) to express the proteins. BL21(Rosetta) cells containing pET28a-RS01790, pET28a-BMP, pET28a-GrpE, pET28a-RS00900, and pET28a-RS00275 were cultured in LB medium with kanamycin at 37 °C until they reached the logarithmic phase (OD_600_ of 0.6). Subsequently, the culture was induced at 37 °C for 4 h with 0.5 mM IPTG. The proteins were purified using High-Affinity Ni-NTA Resin (GenScript, Nanjing, China). The protein concentrations of purified recombinant proteins were quantified using the BCA Protein Assay Kit (Beyotime, Shanghai, China) and analyzed by SDS-PAGE and western blot as described above. Western blot analysis was performed using mouse anti‐His‐tag monoclonal primary antibodies (Boster Biological Technology co.Itd, M30975; 1∶5000) and goat anti-mouse IgG-HRP (H + L) antibody (Boster Biological Technology co.Itd, BA1050; 1∶5000).

### Antigenicity and immunogenicity analysis

To evaluate the antigenicity and immunogenicity of *M. synoviae* proteins expressed in *E. coli*, western blot assays were carried out as described above. Ten micrograms (μg) purified recombinant protein or 10 μg of *M. synoviae* cell lysates was subjected to SDS‐PAGE and transferred onto PVDF membranes. The membranes were incubated separately with mouse sera immunized with one of the five recombinant proteins or rabbit anti-*M. synoviae* polyclonal antibodies (1∶1000). Subsequently, they were incubated with goat anti-mouse or goat anti-rabbit IgG-HRP (H + L) antibody (1∶5000).

### Surface localization and distribution

To evaluate the subcellular localization of the target molecules cytoplasmic, membrane and secretory fractions of *M. synoviae* cells were analyzed by western blot following the previously described method^35^. Western blotting was performed as described above, using five antisera against the recombinant protein (1∶1000) and goat anti-mouse IgG-HRP (H + L) antibody.

The surface localization of RS01790, BMP, GrpE, RS00900 and RS00275 on *M.synoviae* cells was determined using suspension immunofluorescence assays, as previously described in ref. ^50^. Briefly, a 50 mL sample of *M. synoviae* culture in the mid-logarithmic growth phase was collected by centrifugation at 12,000 × *g* for 30 min. The pellet was washed and resuspended in PBS buffer containing 2% (w/v) BSA, followed by incubation for 1 h at 37 °C. After centrifugation incubated with five antisera against the recombinant protein (1∶100 diluted in PBST) at 37 °C for 1 h respectively. Next, the *M. synoviae* samples were incubated with goat anti-mouse IgG-FITC (fluorescein isothiocyanate) antibody (Bioss, Beijing, China, bs-0296G-FITC; 1∶500) at 37 °C for 1 h. After washing, the *M. synoviae* samples were spread onto glass slides and observed by fluorescence microscopy (LSM800; Zeiss, German). Nonimmunized mouse sera were used as negative controls.

### Adherence analysis

To detect the adherence of rRS01790, rBMP, rGrpE, rRS00900 and rRS00275 to DF‐1 cells, an indirect immunofluorescence assay was used as described previously with some modifications^15^. DF-1 cells were cultured on coverslips in 24-well cell culture plates for 24 h. After washing, the DF-1 cells were incubated with 5 µg of freshly purified recombinant antigenic proteins in 500 µL DMEM for 1 hour at 37 °C. DF-1 cells without any protein adhesion were used as controls. After proteins incubation, the cells were washed three times with PBS. DF-1 cells were fixed with 4% PFA (paraformaldehyde) for 15 min and incubated in PBS buffer containing 2% (w/v) BSA for 1 hour at 37 °C. And then labeled with mouse anti‐His‐tag IgG (1∶5000) and goat anti-mouse IgG‐FITC for 1 h respectively. The cell membranes and nuclei were labeled with 10 μM DilC18 (Beyotime) and 0.1 μg/mL DAPI (Beyotime) at room temperature for 10 min, respectively. Finally, the cellular coverslips were treated with an antifade mounting medium (Sangon Biotech, China) and observed under a laser scanning confocal microscope (LSM800; Zeiss, Germany).

### Immunization of chicken

To evaluate the immune protective effect of candidate antigens, experimental one-day-old chickens were randomly divided into 7 groups (*n* = 12 per group). The expressed recombinant proteins were diluted properly with PBS, mixed with MontanideTM ISA 206 VG adjuvant, then emulsified thoroughly to prepare the subunit vaccine. Chickens were immunized intramuscularly (i.m.) in the leg with a prime vaccination on day 0 (one-day-old) followed by two booster vaccinations on days 14 and 21 (15-days-old and 22-days-old), using the vaccine formulation containing 100 μg of each protein (RS01790, BMP, GrpE, RS00900, and RS00275). The control group was injected with PBS mixed with MontanideTM ISA 206 VG adjuvant. Blood samples were collected from vaccinated groups and the blank control group via the wing vein every week until 3 weeks post final immunization. The sera were separated to determine antibody levels.

### Challenge with M. synoviae in chicken

One week after the third immunization, six 29-day-old chickens from each vaccinated group and control group were randomly selected and challenged with broth culture of rSC0200 (9 × 10^7^ CCU/mL) via tracheal and foot pad injection. The number of CCU (color-changing units) was assessed as previously described in ref. ^51^. Briefly, a ten-fold dilution series (up to 10^–9^) of *M. synoviae* strain culture in Frey’s medium was prepared. Samples that showed no color change after 2 weeks at 37 °C were considered negative for *M. synoviae* colonization. The CCU titer was expressed as the mean CCU/mL. The infection details were as follows: challenged chickens were injected with 100 μL into the trachea and 100 μL into the left foot pads. The negative control group received injections of Frey’s broth. To increase the incidence and severity of mycoplasma lesions, NDV/IBV live nasal vaccine (30 μL per bird) was administered.

The chickens were individually weighed on the day of primovaccination (day 0, one-day-old), challenge (days 28, 29-days-old), and euthanasia (days 49, 50-days-old). The average daily weight gain (ADWG; grams/day) was calculated from day 0 to days 28 and from days 28 to days 49. ADWG was computed as the difference between the initial and final weight, divided by the duration of the stage. Gross air sac lesions were scored on a scale of 0 to 4 at necropsy, using a modification of a system described previously in ref. ^52^. Tracheal tissues from the upper, middle, and lower regions were collected for histopathological examination, as previously described in ref. ^53^. Briefly, tissue sections were fixed in 10% neutral buffered formalin for a minimum of 24 h, dehydrated, embedded in paraffin wax, sectioned (at a thickness of 5 μm), and stained with hematoxylin and eosin. To determine the average mucosal thickness, four different points on the vertical and horizontal lines were measure. Laryngeal swabs were obtained for quantitative real-time PCR (qRT-PCR). The experimental design is shown in Fig. 5a.

### Lymphocyte proliferation assay

CCK‐8 assays were performed to determine lymphocyte proliferation, following a previously described method with some modifications^54^. In brief, peripheral blood mononuclear cells (PBMCs) were isolated from blood samples collected from chickens at two weeks post final immunization using a chicken PBMC isolation kit (Solarbio, Beijing, China). The PBMCs were cultured in RPMI 1640 medium at a density of 2 × 10^5^ cells per well in 96-well plates and maintained in a humidified incubator at 37 °C and 5% CO_2_ for 48 h. During the incubation, the cells were stimulated with either antigenic proteins or Concanavalin A (ConA). The antigenic proteins were diluted with RPMI 1640 medium to a concentration 20 μg/mL and added to PBMCs. Cells without any treatment served as a blank control referred to as 1640. After the 48-hour incubation period, 10 μL of CCK-8 reagent (Solarbio, Beijing, China) was added to each well, and the plates were further incubated at 37 °C for 3 h. The optical density (OD) was then measured at 450 nm using an automatic microplate reader. The stimulation index (SI) was calculated as the ratio of the OD of antigen-stimulated cells to that of unstimulated cells.

### Quantification of cytokine genes expression

PBMCs were cultured at a density of 1 × 10^7^ per well in 24‐well plates and maintained in a humidified incubator at 37 °C and 5% CO_2_ for 48 h with either antigenic proteins or ConA stimulation, using the same concentration as described above. The mRNA expression levels of IFN‐γ, IL‐17 A, IL‐4 and IL‐6 in PBMCs were detected by qRT-PCR. Total RNA was extracted using the Fast Pure Cell/Tissue Total RNA Isolation Kit (Vazyme Biotech Co., Ltd). The purified RNA was reverse transcribed into cDNA using the PrimeScript™ RT Master Mix (TaKaRa, Beijing, China). qRT-PCR was performed using the Taq Pro Universal SYBR qPCR Master Mix (Vazyme Biotech Co., Ltd). Amplification procedures were carried out according to the instructions: pre-denaturation at 95 °C for 30 s, followed by 40 cycles of denaturation at 95°C for 10 seconds and extension at 60 °C for 30 s, and a melting curve analysis was performed at the end of the qRT-PCR. The primers are shown in Table 2. Gene expression levels were analyzed using the 2^‐ΔΔct^ method, with β-actin serving as a housekeeping gene^53,55^.

**Table 2:** The primers information.

Primer name	Sequences (5’–3’)	References
MS-qPCR-F	ATTGCTTGTGCTAGCGTTTATCC	^56^
MS-qPCR-R	ATTTGGTGGCGCTAAATTAACC	
MS-plasmid-F	CTTACTAGTTTCAGCGCTCTT	This study
MS-plasmid-R	ATATTAGCGCTTAGTTTATTTTCTA	This study
pET-28a-F	TAATACGACTCACTATAGGG	This study
pET-28a-R	GCTAGTTATTGCTCAGCGG	This study
β-actin-F	CAACACAGTGCTGTCTGGTGG	^55^
β-actin-R	ATCGTACTCCTGCTTGCTGATCC	
IL-17A-F	CTCCGATCCCTTATTCTCCTC	^53^
IL-17A-R	AAGCGGTTGTGGTCCTCAT	
IFN-γ-F	AGCTGACGGTGGACCTATTATT	
IFN-γ-R	GGCTTTGCGCTGGATTC	
IL-4-F	TGAATGACATCCAGGGAGAG	
IL-4-R	GGCTTTGCATAAGAGCTCAG	
IL-6-F	CAAGGTGACGGAGGAGGAC	
IL-6-R	TGGCGAGGAGGGATTTCT	

### Quantification of *M. synoviae* DNA

Total DNA from collected laryngeal swabs was extracted using Hi-Swab DNA Kit (TIANGEN Biotech Co., Ltd) according to the manufacturer’s instructions. A standard plasmid containing the target sequence from the *M. synoviae* genome was constructed to analyze the data using the absolute quantitative method, following a previously described protocol with some modifications^56^. In brief, to obtain *M. synoviae* partial targeted gene sequences (Supplementary Fig. 13a) that contain the qRT-PCR primers, we conducted standard PCR using the primers listed in Table 2. The amplified products were cloned into the pMD-19T vector (TaKaRa, Japan) according to the user manual and transformed into *E. coli* DH5α cells. The number of recombinant plasmids was determined using an online calculator provided by the URI Genomics & Sequencing Center (http://cels.uri.edu/gsc/cndna .html) for estimating the number of copies of a template. To create a standard curve, we performed serial 1∶10 dilutions of purified *M. synoviae* plasmid (Supplementary Fig. 13b). Using the resulting curve, we calculated the absolute copy numbers of total bacteria. Amplification procedures were carried out according to the described above. The primers are shown in Table 2.

### Statistical analyses

Data were compared using Mann Whitney U Test. When *p* < 0.05 the differences were considered significant. GraphPad Prism was used to analyze data, which were shown as the mean ± SD.

### Reporting summary

Further information on research design is available in the Nature Research Reporting Summary linked to this article.

### Supplementary information

Supplementary Information

Reporting Summary

## Data Availability

All data generated or analyzed during this study are presented in the article, and materials are available from the corresponding author upon reasonable request.
