# Supplementary material for: Screening of immunogenic proteins and evaluation of vaccine candidates against Mycoplasma synoviae
Source: NPJ Vaccines. 2023 Aug 15;8:121. doi: 10.1038/s41541-023-00721-y (PMC10427712; doi:10.1038/s41541-023-00721-y)
Supplement: Supplementary file 1 — Supplementary Information [file 41541_2023_721_MOESM1_ESM.pdf]

SUPPLEMENTARY FIGURES

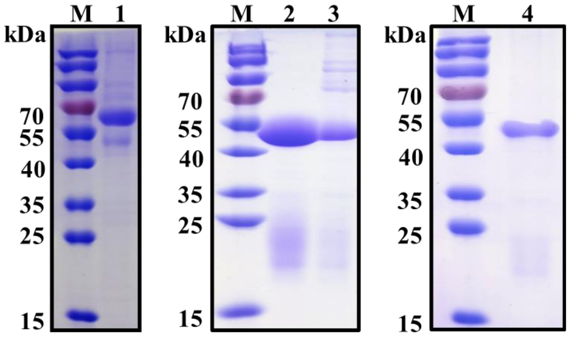

Supplementary Figure 1. SDS-PAGE analysis of purified rabbit anti-*M. synoviae* IgG. M, protein marker; Line 1, rabbit serum before purification; Line 2, Purified anti-*M. synoviae* IgG using protein A/G affinity chromatogram; Line 3, Purified rabbit anti-*M. synoviae* IgG using Ammonium sulphate; Line 4, Rabbit anti-*M. synoviae* IgG dialyzed into PBS after protein A/G affinity chromatogram purification.

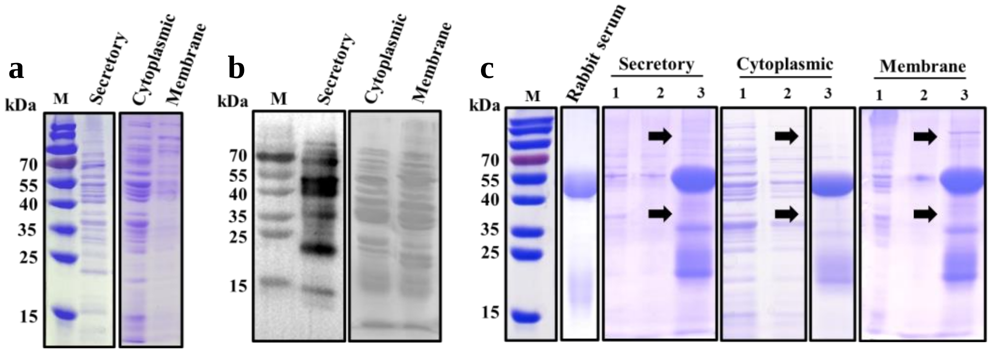

Supplementary Figure 2. Extraction and immunoprecipitation analysis of cytoplasmic protein, membrane protein and secretory protein of *M. synoviae*. (a) SDS-PAGE analysis of *M. synoviae* cytoplasmic protein, membrane protein and secretory protein extraction. (b) Western blot analysis of cytoplasmic protein, membrane protein and secretory protein of *M. synoviae.* (c) Immunoprecipitation analysis of *Mycoplasma synoviae* proteins. Line 1, input sample; Line 2, flow through sample; Line 3, elution sample. Arrowheads indicated immunogenic proteins.

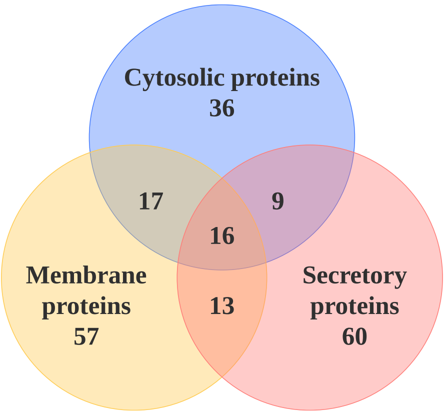

Supplementary Figure 3. Localization of LC-MS/MS analysis identified proteins.

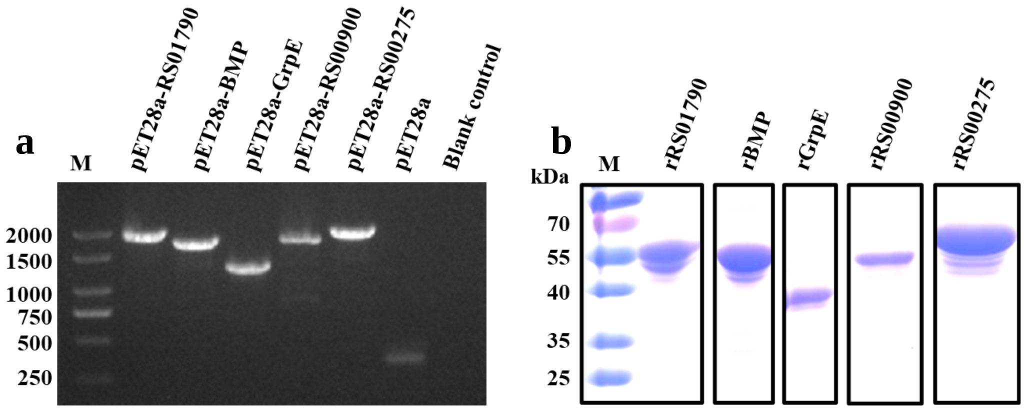

Supplementary Figure 4. Cloning, expression and purification of *M. synoviae* RS01790, BMP, GrpE, RS00900 and RS00275 proteins. (a)PCR detection of expression plasmid using T7 promoter and terminator primers. M, 2-kb DNA ladder. (b) SDS-PAGE analysis of purified recombinant proteins using High-Affinity Ni-NTA Resin.

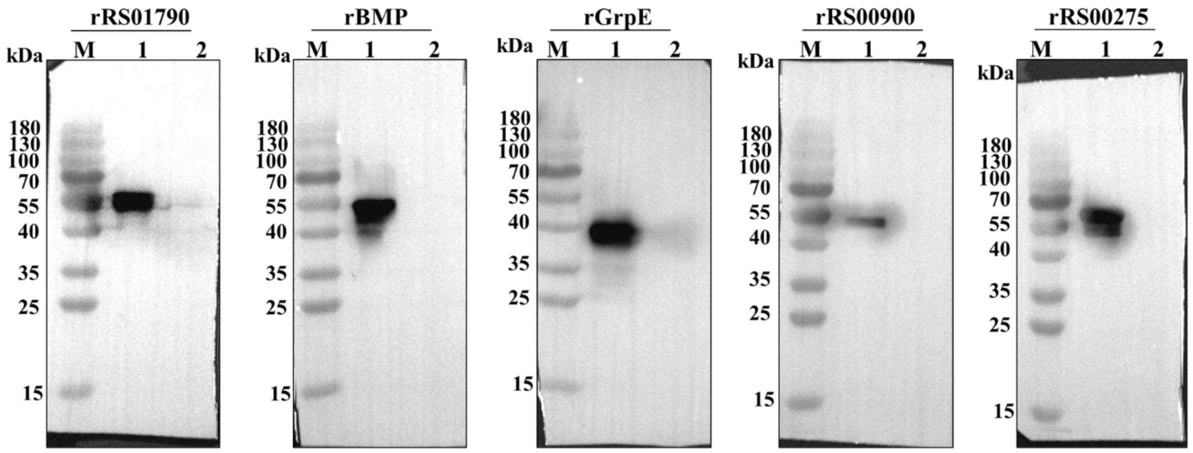

Supplementary Figure 5. Original blots of of purified recombinant protein.
M, protein marker; Lane 1, recombinant proteins; Lane 2, total-cell lysate of *E. coli* pET28a.

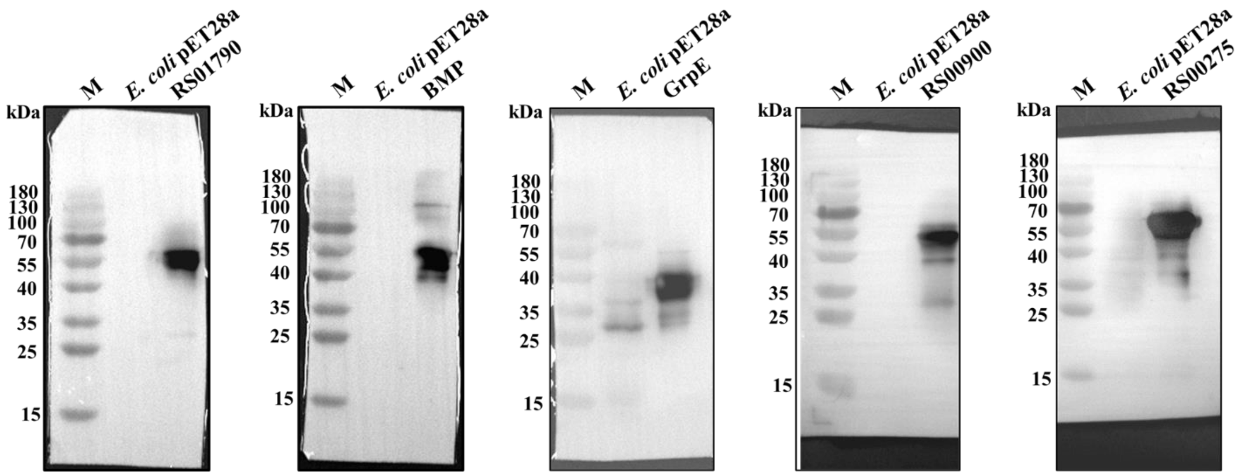

Supplementary Figure 6. Original blots of of purified recombinant protein. Incubated with mouse anti – His IgG.

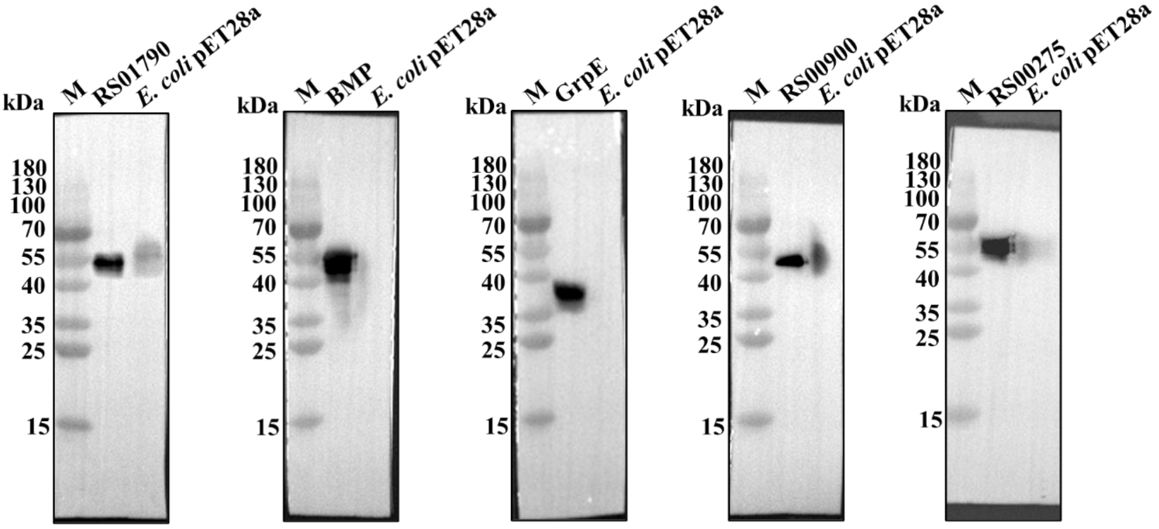

Supplementary Figure 7. Original blots of of purified recombinant protein. Incubated with rabbit anti- *M. synoviae* IgG.

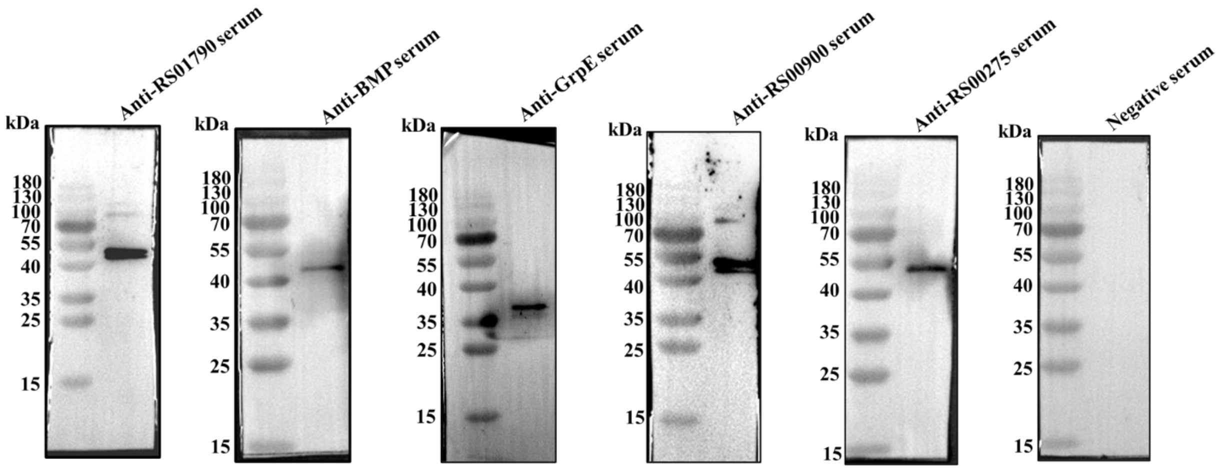

Supplementary Figure 8. Original blots of *M. synoviae* cells. Incubated with the serum of mice immunized with rRS01790, rBMP, rGrpE, rRS00900, and rRS00275, respectively.

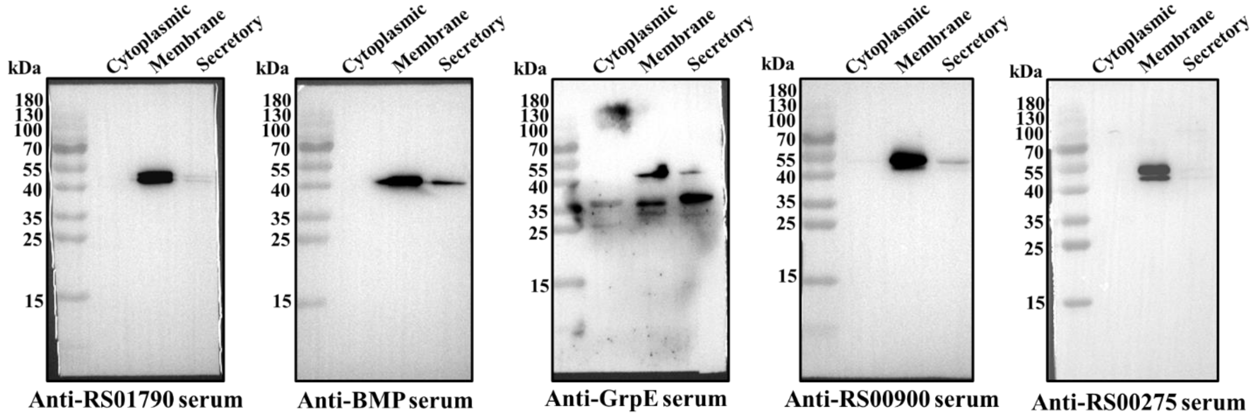

Supplementary Figure 9. Original blots of the distribution of *M. synoviae* RS01790, BMP, GrpE, RS00900, and RS00275. Incubated with the serum of mice immunized with rRS01790, rBMP, rGrpE, rRS00900, and rRS00275, respectively.

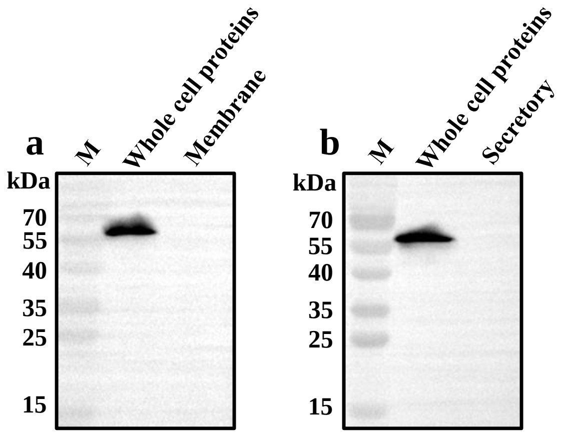

Supplementary Figure 10. Western blot analysis of *M. synoviae* whole cell proteins membrane (a), and secretory (b) proteins, anti-GroEL was used as the primary antibody.

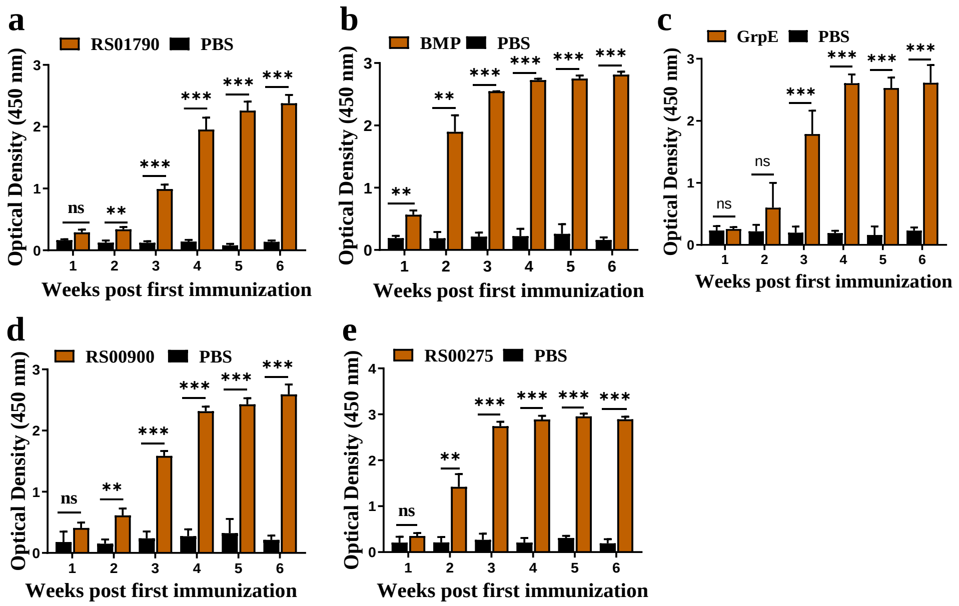

Supplementary Figure 11. Effect of RS01790, BMP, GrpE, RS00900 and RS00275 vaccination on IgG antibody production. Six groups of SPF chicken were immunized intramuscularly with PBS, RS01790 (a), BMP (b), GrpE (c), RS00900 (d) and RS00275 (e). Specific antibody levels in sera were assessed by ELISA. Statistical analysis was performed using the Mann–Whitney U test (***$p < 0.001$, ** $p < 0.01$, * $p < 0.05$, ns $p > 0.05$). Error bar, mean ± S.D.

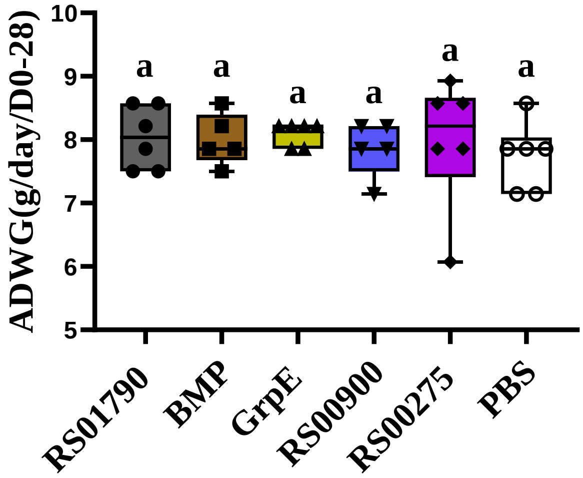

Supplementary Figure 12. Results of average daily weight gain (ADWG) in chickens from day 0 to days 28. Statistical analysis was performed using the Mann–Whitney U test (***$p < 0.001$, ** $p < 0.01$, * $p < 0.05$, ns $p > 0.05$). Error bar, mean ± S.D.

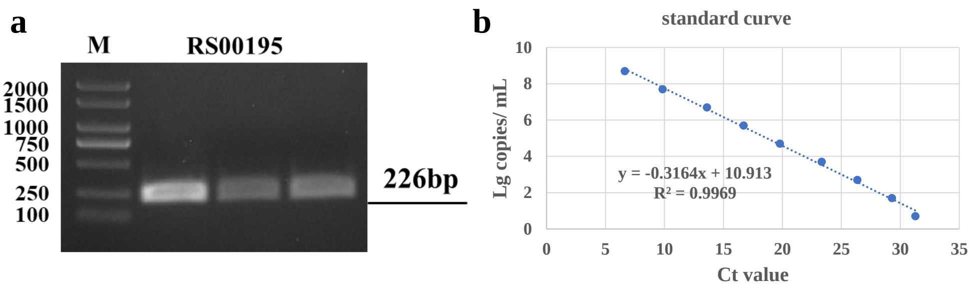

Supplementary Figure 13. Standard curve preparation. (a) PCR of the RS00195 amplified gene, M 2000bp DNA ladder. (b)Standard curve of the real-time PCR based on serial dilutions of the plasmid DNA.

SUPPLEMENTARY TABLE

Supplementary Table 1. Homology of screened immunogenic proteins in different strains

Strains	RS01790	BMP	GrpE	RS00900	RS00275
HN01	100.00%	100.00%	100.00%	100.00%	100.00%
AS2	98.26%	98.88%	99.66%	98.00%	98.59%
18DW	98.07%	98.88%	99.66%	99.78%	98.19%
MS-H	98.26%	98.88%	99.66%	98.00%	98.59%
WF18	100.00%	99.11%	99.32%	100.00%	100.00%
51SH	98.07%	98.88%	99.66%	99.78%	98.19%
ZX313	100.00%	100.00%	99.66%	100.00%	97.59%
BS4S2	97.94%	98.88%	99.66%	99.78%	98.19%
FJ-01	100.00%	100.00%	100.00%	100.00%	97.59%
5-9	100.00%	99.78%	100.00%	100.00%	97.59%
G3	98.07%	98.88%	99.66%	99.33%	98.19%
A4	98.07%	98.88%	99.66%	99.33%	98.19%
86079-7NS	98.26%	98.88%	99.66%	98.00%	98.59%
53	98.45%	99.31%	98.99%	98.45%	97.99%
NCTC10124	98.07%	99.55%	98.99%	98.87%	98.59%
WVU1853	98.07%	99.55%	98.97%	98.87%	98.59%
SD2	98.26%	100.00%	99.32%	100.00%	100.00%
TS4	98.26%	98.88%	99.66%	98.00%	98.59%
AB1	98.26%	98.88%	99.66%	98.00%	98.59%
