# Supplementary material for: Screening of immunogenic proteins and evaluation of vaccine candidates against Mycoplasma synoviae
Source: NPJ Vaccines. 2023 Aug 15;8:121. doi: 10.1038/s41541-023-00721-y (PMC10427712; doi:10.1038/s41541-023-00721-y)
Supplement: Supplementary file 2 — Reporting Summary [file 41541_2023_721_MOESM2_ESM.pdf]

nature portfolio

Corresponding author(s): Huoying Shi

Last updated by author(s): Jul 5, 2023

Reporting Summary

Nature Portfolio wishes to improve the reproducibility of the work that we publish. This form provides structure for consistency and transparency in reporting. For further information on Nature Portfolio policies, see our Editorial Policies and the Editorial Policy Checklist.

Statistics

For all statistical analyses, confirm that the following items are present in the figure legend, table legend, main text, or Methods section.

n/a	Confirmed	
☐	☒	The exact sample size (*n*) for each experimental group/condition, given as a discrete number and unit of measurement
☐	☒	A statement on whether measurements were taken from distinct samples or whether the same sample was measured repeatedly
☐	☒	The statistical test(s) used AND whether they are one- or two-sided<br>*Only common tests should be described solely by name; describe more complex techniques in the Methods section.*
☐	☒	A description of all covariates tested
☐	☒	A description of any assumptions or corrections, such as tests of normality and adjustment for multiple comparisons
☐	☒	A full description of the statistical parameters including central tendency (e.g. means) or other basic estimates (e.g. regression coefficient) AND variation (e.g. standard deviation) or associated estimates of uncertainty (e.g. confidence intervals)
☐	☒	For null hypothesis testing, the test statistic (e.g. F, t, r) with confidence intervals, effect sizes, degrees of freedom and P value noted<br>*Give P values as exact values whenever suitable.*
☒	☐	For Bayesian analysis, information on the choice of priors and Markov chain Monte Carlo settings
☒	☐	For hierarchical and complex designs, identification of the appropriate level for tests and full reporting of outcomes
☐	☒	Estimates of effect sizes (e.g. Cohen's *d*, Pearson's *r*), indicating how they were calculated

Our web collection on statistics for biologists contains articles on many of the points above.

Software and code

Policy information about availability of computer code

Data collection	No software was used.
Data analysis	Vaxign2 is publicly accessible at http://www.violinet.org/vaxign2.<br>Protparam server is publicly accessible at https://web.expasy.org/protparam/.<br>TMHMM (v2.0) server is publicly accessible at https://services.healthtech.dtu.dk/services/TMHMM-2.0/.

For manuscripts utilizing custom algorithms or software that are central to the research but not yet described in published literature, software must be made available to editors and reviewers. We strongly encourage code deposition in a community repository (e.g. GitHub). See the Nature Portfolio guidelines for submitting code & software for further information.

Data

Policy information about availability of data

All manuscripts must include a data availability statement. This statement should provide the following information, where applicable:

- Accession codes, unique identifiers, or web links for publicly available datasets
- A description of any restrictions on data availability
- For clinical datasets or third party data, please ensure that the statement adheres to our policy

Accession codes, unique identifiers, or web links for publicly available datasets.

Research involving human participants, their data, or biological material

Policy information about studies with human participants or human data. See also policy information about sex, gender (identity/presentation), and sexual orientation and race, ethnicity and racism.

Reporting on sex and gender	This study does not involve the human participants.
Reporting on race, ethnicity, or other socially relevant groupings	This study does not involve the human participants.
Population characteristics	This study does not involve the human participants.
Recruitment	This study does not involve the human participants.
Ethics oversight	This study does not involve the human participants.

Note that full information on the approval of the study protocol must also be provided in the manuscript.

Field-specific reporting

Please select the one below that is the best fit for your research. If you are not sure, read the appropriate sections before making your selection.

☒ Life sciences ☐ Behavioural & social sciences ☐ Ecological, evolutionary & environmental sciences

For a reference copy of the document with all sections, see nature.com/documents/nr-reporting-summary-flat.pdf

Life sciences study design

All studies must disclose on these points even when the disclosure is negative.

Sample size	Five BALB/c mice were used to prepare polyclonal antibodies against each recombinant protein. Three New Zealand white rabbits were used to prepare polyclonal antibodies against chicken synovial bursa. Twelve SPF chickens were used to study the immune response and protective efficiency of subunit vaccines.
Data exclusions	No dota were excluded from the analyses
Replication	All attempts at replication were successful
Randomization	Allocation was random
Blinding	Investigators were blinded to group allocotion during data collection and/or analysis.

Reporting for specific materials, systems and methods

We require information from authors about some types of materials, experimental systems and methods used in many studies. Here, indicate whether each material, system or method listed is relevant to your study. If you are not sure if a list item applies to your research, read the appropriate section before selecting a response.

Materials & experimental systems

n/a	Involved in the study	
☐	☒	Antibodies
☐	☒	Eukaryotic cell lines
☒	☐	Palaeontology and archaeology
☐	☒	Animals and other organisms
☒	☐	Clinical data
☒	☐	Dual use research of concern
☒	☐	Plants

Methods

n/a	Involved in the study	
☒	☐	ChIP-seq
☒	☐	Flow cytometry
☒	☐	MRI-based neuroimaging

Antibodies

Antibodies used	anti-His-tag monoclonal primary antibodies (Boster Biological Technology co.ltd, M30975)；<br>rabbit anti-groEL antibody (Abcom, ab90522)

Validation	https://www.bosterbio.com/antibodies-validation-information<br>https://www.abcam.cn/products/primary-antibodies/groel-antibody-ab90522.html

Eukaryotic cell lines

Policy information about cell lines and Sex and Gender in Research

Cell line source(s)	The DF-1 chicken embryo fibroblast cells were obtained from ATCC.
Authentication	UMNSAH/DF-1(CRL-3586, chicken embryonic fibroblasts) is a spontaneously immortalized chicken cell line. This product is an ATCC manufactured and accessioned progeny of ATCC CRL-12203 cited in US Pat. No. 5,672,485.
Mycoplasma contamination	All cell lines tested negative for mycoplasma contamination
Commonly misidentified lines (See ICLAC register)	No commonly misidentified cell lines used in the study

Animals and other research organisms

Policy information about studies involving animals; ARRIVE guidelines recommended for reporting animal research, and Sex and Gender in Research

Laboratory animals	Female six-week-old BALB/c mice were purchased from the Comparative Medicine Center, Yangzhou University (Jiangsu, China). Female two-month-old New Zealand white rabbits (Shanghai Songlian Experimental Animal Farm, Shanghai, China). The specific pathogen-free (SPF) chicken embryos were purchased from Nanjing Biology Medical Factory, Qian Yuan-hao Biological Co., Ltd.
Wild animals	This study did not involve wild animals.
Reporting on sex	The results of this study not only used one gender, but also used female mice and female rabbits to prepare polyclonal antibodies in the study because the female character was gentle and convenient for blood collection.
Field-collected samples	The study did not involve samples collected from the field.
Ethics oversight	Animals were euthanized by exsanguination under anesthesia.

Note that full information on the approval of the study protocol must also be provided in the manuscript.
